# LPI-HyADBS: a hybrid framework for lncRNA-protein interaction prediction integrating feature selection and classification

**DOI:** 10.1186/s12859-021-04485-x

**Published:** 2021-11-26

**Authors:** Liqian Zhou, Qi Duan, Xiongfei Tian, He Xu, Jianxin Tang, Lihong Peng

**Affiliations:** 1 School of Computer, Hunan University of Technology, Zhuzhou, China; 2 College of Life Sciences and Chemistry, Hunan University of Technology, Zhuzhou, China

**Keywords:** *C*-SVM, Deep neural network, Ensemble learning, Feature selection, lncRNA-protein interaction, XGBoost

## Abstract

**Background:**

Long noncoding RNAs (lncRNAs) have dense linkages with a plethora of important cellular activities. lncRNAs exert functions by linking with corresponding RNA-binding proteins. Since experimental techniques to detect lncRNA-protein interactions (LPIs) are laborious and time-consuming, a few computational methods have been reported for LPI prediction. However, computation-based LPI identification methods have the following limitations: (1) Most methods were evaluated on a single dataset, and researchers may thus fail to measure their generalization ability. (2) The majority of methods were validated under cross validation on lncRNA-protein pairs, did not investigate the performance under other cross validations, especially for cross validation on independent lncRNAs and independent proteins. (3) lncRNAs and proteins have abundant biological information, how to select informative features need to further investigate.

**Results:**

Under a hybrid framework (LPI-HyADBS) integrating feature selection based on AdaBoost, and classification models including deep neural network (DNN), extreme gradient Boost (XGBoost), and SVM with a penalty Coefficient of misclassification (*C*-SVM), this work focuses on finding new LPIs. First, five datasets are arranged. Each dataset contains lncRNA sequences, protein sequences, and an LPI network. Second, biological features of lncRNAs and proteins are acquired based on Pyfeat. Third, the obtained features of lncRNAs and proteins are selected based on AdaBoost and concatenated to depict each LPI sample. Fourth, DNN, XGBoost, and *C*-SVM are used to classify lncRNA-protein pairs based on the concatenated features. Finally, a hybrid framework is developed to integrate the classification results from the above three classifiers. LPI-HyADBS is compared to six classical LPI prediction approaches (LPI-SKF, LPI-NRLMF, Capsule-LPI, LPI-CNNCP, LPLNP, and LPBNI) on five datasets under 5-fold cross validations on lncRNAs, proteins, lncRNA-protein pairs, and independent lncRNAs and independent proteins. The results show LPI-HyADBS has the best LPI prediction performance under four different cross validations. In particular, LPI-HyADBS obtains better classification ability than other six approaches under the constructed independent dataset. Case analyses suggest that there is relevance between ZNF667-AS1 and Q15717.

**Conclusions:**

Integrating feature selection approach based on AdaBoost, three classification techniques including DNN, XGBoost, and *C*-SVM, this work develops a hybrid framework to identify new linkages between lncRNAs and proteins.

**Supplementary Information:**

The online version contains supplementary material available at 10.1186/s12859-021-04485-x.

## Introduction

### Motivation

RNA-protein interactions regulate many cellular processes including splicing, polyadenylation, stability, transportation and translation [1, 2]. Recently, an increasing knowledge about RNA-binding proteins is shifting towards long non-coding RNAs (lncRNAs) [3, 4]. lncRNAs are a class of transcribed RNA molecules with the length of more than 200 nucleotides [5, 6]. The class of molecules are densely associated with a plethora of cellular activities and play vital roles in regulating gene expression [7]. The dysregulations of lncRNAs may result in various diseases, particularly cancers [8, 9]. For example, lncRNA-protein complex may influence severity degree of human pancreatic cancer phenotype. lncRNAs have been validated to closely link with poorer prognosis in lymphoma, colon cancer, and breast cancer [10].

Despite of abundant information about lncRNA-disease associations, their mechanisms still remain enigmatic. Researches found that lncRNAs exert their regulation roles through associations with the homologous RNA-binding proteins, that is, lncRNA-protein interactions (LPIs) [10–12]. Therefore, identification of LPIs will be beneficial to complex disease research and can thus advance diagnosis and treatment procedures [11]. Considering the time-consuming and laborious nature of laboratory methods, researchers pay more attention to computational intelligence [13]. Computation methods for LPI prediction can be roughly grouped into two categories: network-based approaches and machine learning-based approaches.

Network-based approaches took advantage of known LPIs to find unknown LPIs [14–16]. Li et al. [17] explored a random walk with restart algorithm (LPIHN) to propagate labels of LPIs on a heterogeneous lncRNA-protein network. Ge et al. [18] used a two-step algorithm (LPBNI) on a bipartite network. Hu et al. [19] delineated a semi-supervised lncRNA-protein linkage inference framework called LPI-ETSLP. Deng et al. [20] integrated diffusion and HeteSim features on the heterogeneous lncRNA-protein network (PLIPCOM). Zheng et al. [21] fused sequences, domains, GO terms of proteins and the STRING database and built a more informative model. Zhang et al. [22] proposed a linear neighborhood propagation method (LPLNP) for LPI mining. Zhou et al. [23] developed a similarity kernel fusion-based algorithm, LPI-SKF. Zhang et al. [24] adopted a network distance analysis technique. Network-based approaches uncovered many linkages between lncRNAs and proteins, however, they are out of the LPI prediction problem for a new lncRNA or protein.

Machine learning-based approaches including ensemble learning-based approaches [25–27] and deep learning-based approaches have increasingly achieved more attentions. Muppirala et al. [28] combined support vector machine (SVM) and random forest and proposed an LPI identification algorithm (RPISeq). Wang et al. [29] used an extended naive Bayes model to find hidden LPIs. Suresh et al. [30] built an SVM-based LPI inference model with sequence and structure information. Zhao et al. [31] and Liu et al. [32] proposed two neighborhood regularized matrix factorization-based methods, IRWNRLPI and LPI-NRLMF. Hu et al. [19] adopted an eigenvalue transformation-based semi-supervised LPI prediction approach.

Ensemble learning-based models demonstrated powerful performance in various association prediction area [26]. Zhang et al. [33] designed a sequence feature projection-based ensemble learning framework for predicting LPIs. Hu et al. [19] adopted an ensemble strategy for LPI discovery. Wekesa et al. [34] combined an innovative feature selection technique and an ordered boosting algorithm [35] (LPI-XGBoost) to mine new LPIs. Yi et al. [36] presented a learning distributed representation algorithm based on RNA and protein sequences.

Deep learning has been widely applied to capture unobserved LPIs and obtained remarkable performance [37]. Pan et al. [38] made use of stacked ensembling model (IPMiner) to mine underlying ncRNA-protein interaction sequential patterns. Zhang et al. [39] designed a hybrid deep learning architecture combining convolutional neural network (CNN) and recurrent neural network for LPI detection. Pan et al. [40] proposed a deep learning-based method (iDeepS) to identify RNA-binding proteins based on CNNs and a bidirectional long short term memory network (Bi-LSTM). Deng et al. [41] presented a deep neural network-based inference framework (PLIPCOM) through distributed representations of RNA sequences and structures. Fan et al. [42] trained a broad learning-based stacked ensemble classifier. Zhang et al. [43] used a CNN combing the copy-padding trick (LPI-CNNCP). Song et al. [44] and Li et al. [45] exploited capsule network-based prediction techniques (AC-caps and Capsule-LPI).

Previous studies significantly searched the interplays between lncRNAs and proteins, however, several problems still remain to solve: (1) The majority of models were measured on one unique dataset, and it is difficult to investigate their generalization performance. (2) Most algorithms were validated the prediction performance based on Cross Validation (CV) on lncRNA-protein pairs, fail to report the measurements under other CVs, for example, CVs on lncRNAs, proteins, and independent lncRNAs and independent proteins. (3) There are abundant biological information about lncRNAs and proteins. How to effectively integrate these biological characteristics to improve the prediction performance must be considered.

### Study contributions

In this manuscript, a hybrid framework (LPI-HyADBS) is presented to identify LPI candidates. This framework takes advantages of diverse biological information acquisition, feature selection, and ensemble learning. The study has three main contributions:

1. A feature selection algorithm based on AdaBoost is proposed to select the most representative biological features from the originally acquired lncRNA and protein features.
2. A hybrid framework combining deep neural network (DNN), extreme gradient boost (XGBoost), and SVM with a penalty coefficient of misclassification (*C*-SVM) is developed to capture unobserved LPIs.
3. Four different CVs, especially for CV on independent lncRNAs and independent proteins, and five different LPI datasets are applied to further evaluate the generalization ability of the proposed LPI-Hybrid framework.

## Materials and methods

### Data preparation

In this study, we arrange five different LPI datasets. Each dataset contains lncRNA sequences, protein sequences, and an LPI network. Datasets 1, 2, and 3 were from human and were provided by Li et al. [17], Zheng et al. [21], and Zhang et al. [22], respectively. We preprocess the three datasets by removing lncRNAs and proteins involved in one associated protein (or lncRNA) or without sequence or expression information in UniProt [46], NPInter [47], NONCODE [48], and SUPERFAMILY [49]. Datasets 4 and 5 were from plant Arabidopsis thaliana and Zea mays, respectively. The two datasets were provided by Bai et al. [50]. Sequences of lncRNAs and proteins can be achieved from PlncRNADB [50]) and known LPIs can be downloaded from http://bis.zju.edu.cn/PlncRNADB/. The details are shown in Table 1.

**Table 1:** The statistics of LPI data

Dataset	lncRNAs	Proteins	LPIs
Dataset 1	935	59	3479
Dataset 2	885	84	3265
Dataset 3	990	27	4158
Dataset 4	109	35	948
Dataset 5	1704	42	22,133

Each LPI network is defined as a matrix *Y* where

1 $$\begin{aligned} {y_{ij}} = \left\{ {\begin{array}{*{20}{c}} {1,{\mathrm{\, if \,\,lncRNAs \,\,}}{l_i}{\mathrm{\,\, interacts \,\,with \,\,protein\,\, }}{p_j}}\\ {0,{\mathrm{\quad \qquad \qquad \qquad \qquad \qquad \qquad otherwise}}} \end{array}} \right. \end{aligned}$$

### Overview of LPI-HyADBS

**Fig. 1:**
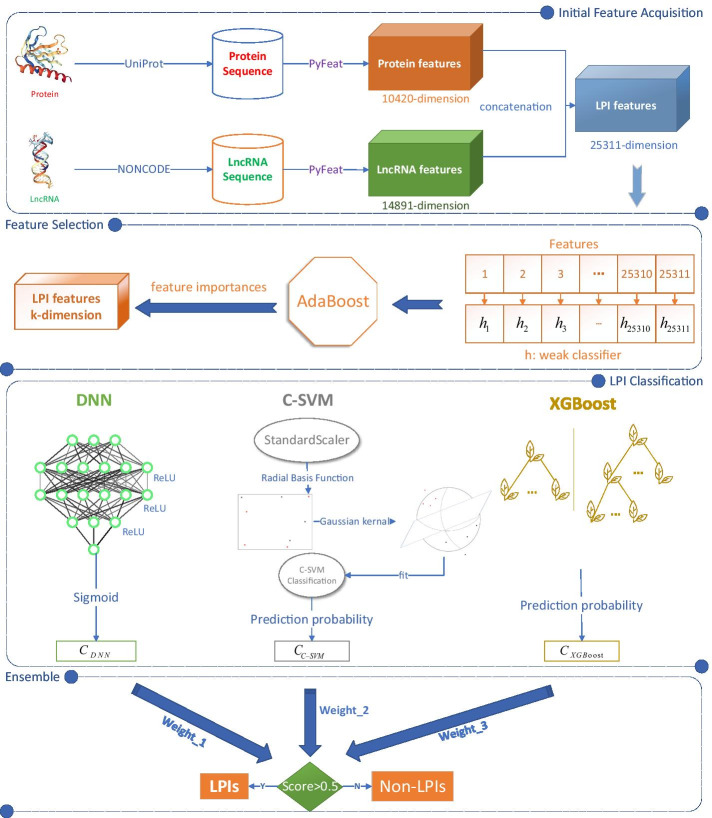
The Pipeline of the LPI-HyADBS framework. (1) Initial feature acquisition. lncRNA and protein features are acquired with Pyfeat [51] and concatenated to depict each lncRNA-protein pair. (2) Feature selection. The concatenated features are reduced based on AdaBoost. (3) LPI classification. DNN, XGBoost, and *C*-SVM are designed to capture unobserved LPIs, respectively. (4) Ensemble. An ensemble framework is proposed to combine prediction results from the three classifiers

In this manuscript, we propose a hybrid framework for LPI identification (LPI-HyADBS). Figure 1 illustrates the pipeline of LPI-HyADBS after data arrangement. As shown in Fig. 1, the LPI-HyADBS method contains the following five procedures: (1) Data arrangement. Five LPI datasets are obtained and preprocessed. Each dataset contains lncRNA sequences, proteins sequences, and an LPI matrix. (2) Initial feature acquisition. lncRNA and protein features are characterized using Pyfeat [51] and concatenated to characterize each lncRNA-protein pair. (3) Feature selection. The concatenated features are reduced based on AdaBoost. (4) LPI classification. DNN, XGBoost, and *C*-SVM are designed to classify unknown lncRNA-protein pairs, respectively. (5) Ensemble. A hybrid framework is developed to integrate the classification results from the three classifiers.

### Initial feature acquisition

Pyfeat [51] is used to acquire initial numerical features of lncRNAs and proteins based on their sequences. We set *k* as 5 in all *k*gap-related features. The obtained lncRNA features include ATGC Ratio (1 feature), CumulativeSkew (2 features), diDiKGap ($256 \times 5=1280$ features), diMonoKGap ($64 \times 5= 320$ features), diTriKGap ($1024 \times 5=5120$ features), gcContent (1 feature), monoDiKGap ($64 \times 5=320$ features), monoMonoKGap ($16 \times 5=80$ features), monoTriKGap ($256 \times 5=1280$ features), Chou’s pseudoKNC (84 features), triMonoKGap ($256 \times 5=1280$ features), tri-DiKGap ($1024 \times 5=5120$ features), and zCurve (3 features). Each lncRNA is represented as a 14,891-dimensional vector based on the above features.

The obtained protein features include pseudoKNC (8420 features) and monoMonoKGap ($400 \times 5=2000$ features). Each protein is denoted as a 10,420-dimensional vector based on the pseudoKNC and monoMonoKGap features.

### Feature selection

Feature selection has been broadly applied to eliminate redundant features and plays an important role in classification. To delete irrelevant features, Gao et al. [52] presented two novel feature selection approaches, that is, linear feature selection method based on class-specific mutual information variation and multilabel feature selection method with constrained latent structure shared term [53]. The two methods obtained the best performance in corresponding application area and are the most representative feature selection techniques.

During the feature acquisition process in the above section, the obtained lncRNA and protein features are highly redundant, which severely increases computational time and affects prediction performance. AdaBoost has good generalization ability, better performance and low computational complexity, and has thus become one of the most effective classifiers [54]. In this manuscript, inspired by the two feature selection methods proposed by Gao et al. [52, 53], we utilize AdaBoost and develop a feature selection algorithm to select the most informative features for lncRNAs and proteins.

Based on initial feature acquisition, the obtained two feature vectors are first concatenated and each lncRNA-protein pair is represented as a 25, 313-dimensional vector $\varvec{x}$. The concatenated vector is then used as the input of the feature selection algorithm to select the representative LPI features. The process can be divided into three parts.

Part I Initialization.

For given *n* LPI samples $\varvec{X}=\{(x_{1}^{1}, x_{1}^{2}, \ldots , x_{1}^{m}),\ldots ,(x_{n}^{1}, x_{n}^{2}, \ldots , x_{n}^{m})\}$ where $x_i^{j}$ denotes the *j*th feature of the *i*th sample and the labels $\varvec{Y}=\{y_{1}, y_{2}, \ldots , y_{n}\}$, the weight coefficient for each LPI sample is initialized: $D(x_{i}^{j})=1 / n$.

Part II Iteration and updating.

At each iteration, conducting the following six steps.

Step 1 For each feature *j*, a weak classifier $h_{j}$ is trained to evaluate its importance.

Step 2 Set the corresponding hypothetical relationship between features and labels: $h_{t}=\{x_{i}^{j} \rightarrow Y\}$.

Step 3 The error corresponding to $D(x_{j}^{i})$ is expressed as Eq. (2):

2 $$\begin{aligned} \varepsilon _{t}=\sum _{i: h_{t}(x_{i}^{j}) \ne y_{i}} D_{t}(x_{i}^{j}) \end{aligned}$$

Step 4 For one feature *f* with a minimum error $\varepsilon _{t}$, delete *f* from initial feature set $\varvec{x}$ and add it to the optimal target feature subset $f_o$ by Eq. (3):

3 $$\begin{aligned} \begin{array}{l} \varvec{x} = \varvec{x} - f\\ f_o = f_o + f \end{array} \end{aligned}$$

Step 5 Update the weight for each weak classifier based on the error from the best classifier $h_{t}$ by Eq. (4):

4 $$\begin{aligned} \beta _{t}=0.5 \times \ln \left( \frac{1-\varepsilon _{t}}{\varepsilon _{t}}\right) \end{aligned}$$

Step 6 Update $D(x_{i}^{j})$ by Eq. (5):

5 $$\begin{aligned} D_{t+1}(x_{i}^{j})=\frac{D_{t}(x_{i}^{j})}{N_{t}} \times \left\{ \begin{array}{ll} e^{-\beta _{t}}, & \text { if } h_{t}(x_{i}^{j})=y_{i} \\ e^{\beta _{t}}, & \text { otherwise } \end{array} \right. \end{aligned}$$

where $N_{t}$ is a regularized constant term satisfying:

6 $$\begin{aligned} \sum \limits _{i=1}^{m} D_{t}(x_{i}^{j})=1 \end{aligned}$$

Part III Normalization of features.

We select the optimal *k* LPI features by iteratively updating LPI descriptions based on the performance from multiple weak classifiers. For the obtained *k* optimal features $F=\{(x_{1}^{1}, x_{1}^{2}, \ldots , x_{1}^{k}),(x_{2}^{1}, x_{2}^{2}, \ldots , x_{2}^{k}), \ldots , (x_{n}^{1}, x_{n}^{2}, \ldots , x_{n}^{k})\}$, we normalize each feature:

7 $$\begin{aligned} {{\tilde{x}}}_i^j = \frac{{x_i^j - \min (x_1^j,x_2^j,\ldots ,x_n^j)}}{{\max (x_1^j,x_2^j,\ldots ,x_n^j) - \min (x_1^j,x_2^j,\ldots ,x_n^j)}} \end{aligned}$$

where $\max (x_1^j,x_2^j,\ldots ,x_n^j)$ and $\min (x_1^j,x_2^j,\ldots ,x_n^j)$ denote the maximum and minimum values in one column, respectively.

To boost the tiny difference between a few classifiers, we used decision trees as weak classifiers based on threshold values. Through ensemble of multiple weak classifiers, the feature selection algorithm based on AdaBoost can add the most appropriate features to the optimal target feature subset.

For a given LPI dataset with *n* LPI examples and the selected *k* LPI features ${\mathcal {D}} = \{({\mathbf {x}}_{i}, y_{i})\}(|{\mathcal {D}}| = n, {\mathbf {x}}_{i} \in {\mathbb {R}}^{k}, y_{i} \in \{+1,-1\})$, we aim to classify unknown lncRNA-protein pairs based on DNN, *C*-SVM, and XGBoost, respectively.

### Deep neural network

To build a standard neural network, researchers utilize neurons to generate real-valued activations and adjust the weights. However, training a neural network needs to take long causal chains in the phase of computation. Therefore, a new training method called layer-wise greedy learning was proposed and marked the birth of deep learning [55]. In contrast to traditional artificial intelligence methods, deep learning techniques have been progressing massively broad application in various areas. Given enough labeled data and appropriate models, the deep learning technologies can more accurately map functions [56].

**Fig. 2:**
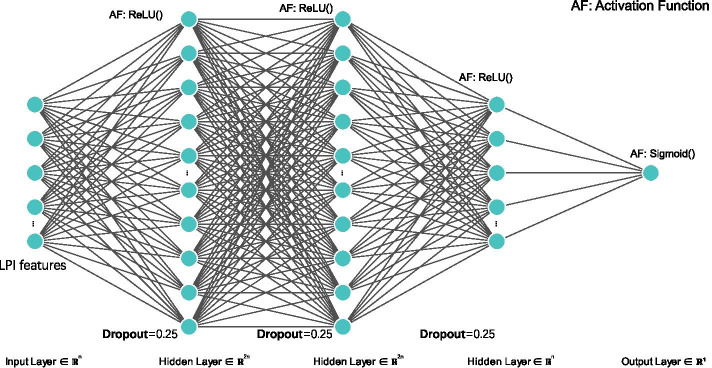
Flowchart of LPI prediction method based on deep neural network

DNNs, employing deep architectures in neural networks, can effectively depict functions with higher complexity when the numbers of layers and neurons in a single layer are increased [57]. DNNs are available to more training data, can improve learning procedures, and demonstrate more computing power and better software engineering [58]. More importantly, it is relatively easy to control overfitting problems during the training of DNNs [59]. Therefore, DNNs have obtained wide applications in various complex machine learning tasks. In this manuscript, the architecture of DNN is illustrated in Fig. 2. It is divided into three main layers, that is, input layer, hidden layers, and output layer. The input layer feeds each LPI sample $\varvec{x}$ into the network. Thus the number of neurons in the input layer is the same as one of the selected LPI features based on AdaBoost. Given an LPI sample $\varvec{x}$, the input layer with *k* inputs is denoted as Eq. (8):

8 $$\begin{aligned} \varvec{x}=[{x}_1,{x}_2,\ldots ,{x}_k] \end{aligned}$$

where $x_i$ denotes the *i*th feature in an LPI sample $\varvec{x}$.

The following layers are the hidden layers. A deep learning framework consists of more than one hidden layer. The hidden layers map each LPI sample $\varvec{x}$ from the input layer. The input in the hidden layers are denoted as Eq. (9):

9 $$\begin{aligned} {h_j} = \sum \limits _{i = 1}^k {{w_i}} {x_i} + {b_j} \end{aligned}$$

where $w_i$ denotes the weight of $x_i$ which are continuing updated to minimize the training errors, *j* indicates the number of hidden layers in the DNN, and $b_j$ denotes the bias in the *j*th hidden layer.

In each hidden layer, there is an activation function. The ReLU function can solve the vanishing and exploding gradient problem, accelerate the training process, and thus demonstrates better performance. Therefore, we use ReLU as an activation function for classifying unlabeled lncRNA-protein pairs.

The output in the *j*th hidden layer are denoted as Eq. (10):

10 $$\begin{aligned} h=f(h_j) \end{aligned}$$

where $$f(h_j)=ReLU(h_j)$$.

Finally, the output layer takes the outputs from the hidden layer as input and produces the output *h* by an activation function. In the output layer, we use sigmoid as an activation function for LPI classification. The output of DNN is represented as Eq. (11):

11 $$\begin{aligned} \sigma (h) = \frac{1}{{1 + {e^{ - h}}}} \end{aligned}$$

An LPI is classified to positive class when the output in the output layer is larger than 0.5; otherwise, the LPI is classified to negative class.

### Extreme gradient boost

XGBoost has high efficiency in both balanced and imbalanced datasets. It is extremely fast due to it parallel computation ability [60]. In known five LPI datasets, there are several positive LPI samples and a large number of unknown lncRNA-protein pairs. That is, known LPI datasets are imbalanced. Considering the imbalanced characteristics of data, we utilize XGBoost to detect underlying LPIs.

#### Regularized learning

Gradient tree boosting techniques obtain widespread applications on the area of bioinformatics [35]. In this study, we use XGBoost to classify unlabeled lncRNA-protein pairs. For a given data set with *n* LPI examples and *k* LPI features ${\mathcal {D}} = \{({\mathbf {x}}_{i}, y_{i})\}(|{\mathcal {D}}| = n, {\mathbf {x}}_{i} \in {\mathbb {R}}^{k}, y_{i} \in \{+1,-1\})$, a tree ensemble model with *M* additive functions can be applied to score each unknown lncRNA-protein pair by Eq. (12).

12 $$\begin{aligned} {\hat{y}}_{i}=\phi ({\mathbf {x}}_{i})=\sum _{j=1}^{M} f_{j}({\mathbf {x}}_{i}), \quad f_{j} \in {\mathcal {F}} \end{aligned}$$

where $f_{j}$ denotes the *j*th tree with structure *q* and leaf weights *w*, ${\mathcal {F}}=\{f({\mathbf {x}})=w_{q({\mathbf {x}})}\}(q: {\mathbb {R}}^{k} \rightarrow T, w \in {\mathbb {R}}^{T})$ indicates the space composed of *k* regression trees, *q* denotes the structure of each tree mapping an LPI sample to corresponding leaf index, and *T* represents the number of leaves in the tree.

For an unknown lncRNA-protein pair, we utilize the decision rules obtained from *q* to compute its final classification result by summing up the interaction scores in the corresponding leaves obtained by *w*. To train the model in Eq. (12), we minimize the following objective function with regularization term by Eq. (13):

13 $$\begin{aligned} \begin{array}{l} {\mathcal {L}}(\phi )=\sum \limits _{i} l({\hat{y}}_{i}, y_{i})+\sum \limits _{j} \Omega (f_{j}) \\ \\ \text { where } \Omega (f)=\gamma T+\frac{1}{2} \lambda \Vert w\Vert ^{2} \end{array} \end{aligned}$$

where *l* denotes a loss function applied to quantify the difference between the predicted label ${\hat{y}}_{i}$ and the real label ${y}_{i}$, and $\Omega$ is used to penalize the complexity of the model. In Eq. (13), the regularization term contributes to reduce overfitting by smoothing the final learned weights. Inspired by the regularized greedy forest model proposed by [61], we set the regularization parameter to zero, and thus the objective function in Eq. (13) is transformed to a gradient tree boosting model.

#### Gradient tree boosting

The model in Eq. (13) is difficult be optimized by the traditional optimization algorithms in Euclidean space. Instead, an additive term is introduced to solve the model Eq. (13). Let ${\hat{y}}_{i}^{(t)}$ denote the predicted label of the *i*th LPI sample at the *t*th iteration, we add $f_{t}$ to the model (13) to minimize the objective function defined by Eq. (14):

14 $$\begin{aligned} {\mathcal {L}}^{(t)}=\sum \limits _{i=1}^{n} l\left( y_{i}, {\hat{y}}_{i}^{(t-1)}+f_{t}({\mathbf {x}}_{i})\right) +\Omega (f_{t}) \end{aligned}$$

By Eq. (14), we gradually add $f_{t}$ to improve the classification capability. The second-order approximation algorithm [62] can be then applied to optimize the model (14) by Eq. (15):

15 $$\begin{aligned} {\mathcal {L}}^{(t)} \simeq \sum \limits _{i=1}^{n}\left[ l\left( y_{i}, {\hat{y}}^{(t-1)}\right) +g_{i} f_{t}({\mathbf {x}}_{i})+\frac{1}{2} h_{i} f_{t}^{2}({\mathbf {x}}_{i})\right] +\Omega (f_{t}) \end{aligned}$$

where $g_{i}=\partial _{{\hat{y}}^{(t-1)}} l(y_{i}, {\hat{y}}^{(t-1)})$ and $h_{i}=\partial _{{\hat{y}}^{(t-1)}}^{2} l(y_{i}, {\hat{y}}^{(t-1)})$ denote first-order and second-order gradient statistics on the cost function, respectively. A simplified objective function denoted by Eq. (16) can be obtained after removing the constant terms at step *t*:

16 $$\begin{aligned} \begin{array}{c} \tilde{{\mathcal {L}}}^{(t)}=\sum \limits _{i=1}^{n}\left[ g_{i} f_{t}({\mathbf {x}}_{i})+\frac{1}{2} h_{i} f_{t}^{2}({\mathbf {x}}_{i})\right] +\Omega (f_{t}) \end{array} \end{aligned}$$

Let $I_{j}=\{i \mid q({\mathbf {x}}_{i})=j\}$ indicate LPI sample set in leaf *j*, Eq. (16) can be rewritten as Eq. (17) by expanding $\omega$:

17 $$\begin{aligned} \tilde{{\mathcal {L}}}^{(t)}&= \sum \limits _{i=1}^{n}\left[ g_{i} f_{t}({\mathbf {x}}_{i})+\frac{1}{2} h_{i} f_{t}^{2}({\mathbf {x}}_{i})\right] +\gamma T+\frac{1}{2} \lambda \sum \limits _{j=1}^{T} w_{j}^{2} \\&= \sum \limits _{j=1}^{T}\left[ \left( \sum \limits _{i \in I_{j}} g_{i}\right) w_{j}+\frac{1}{2}\left( \sum \limits _{i \in I_{j}} h_{i}+\lambda \right) w_{j}^{2}\right] +\gamma T \end{aligned}$$

For a fixed structure $q({\mathbf {x}})$, the optimal weight $w_{j}^{*}$ in leaf *j* can be defined by Eq. (18):

18 $$\begin{aligned} w_{j}^{*}=-\frac{\sum _{i \in I_{j}} g_{i}}{\sum _{i \in I_{j}} h_{i}+\lambda } \end{aligned}$$

and corresponding optimal value can be computed to evaluate the quality of a structure *q* by Eq. (19):

19 $$\begin{aligned} \tilde{{\mathcal {L}}}^{(t)}(q)=-\frac{1}{2} \sum \limits _{j=1}^{T} \frac{\left( \sum _{i \in I_{j}} g_{i}\right) ^{2}}{\sum _{i \in I_{j}} h_{i}+\lambda }+\gamma T \end{aligned}$$

However, it is difficult to enumerate all potential tree structures. We thus use a greedy algorithm to iteratively add branches to a tree starting from a single leaf. Let $I=I_{L} \cup I_{R}$ where $I_L$ and $I_R$ denote LPI sample sets on left and right nodes of a tree after splitting, respectively, we build the loss reduction by Eq. (20):

20 $$\begin{aligned} {\mathcal {L}}_{\text {split }}=\frac{1}{2}\left[ \frac{\left( \sum _{i \in I_{L}} g_{i}\right) ^{2}}{\sum _{i \in I_{L}} h_{i}+\lambda }+\frac{\left( \sum _{i \in I_{R}} g_{i}\right) ^{2}}{\sum _{i \in I_{R}} h_{i}+\lambda }-\frac{\left( \sum _{i \in I} g_{i}\right) ^{2}}{\sum _{i \in I} h_{i}+\lambda }\right] -\gamma \end{aligned}$$

### *C*-support vector machine

SVM is independent of feature dimensionality of data and thus avoids from “curse of dimensionality”. It has better robustness against variation of all vectors except for its support vectors [63]. Considering that the powerful classification ability of SVM, in this section, we utilize *C*-SVM to capture unobserved LPIs.

Given a LPI training dataset $X=\{\varvec{x}_1, \varvec{x}_2,\ldots , \varvec{x}_n\}$ where each LPI sample ${\varvec{x}}_{i} \in R^{k}$, and a label dataset ${\varvec{y}} \in R$ where $\varvec{y}_{i} \in \{1,-1\}$, we use an *C*-SVM provided by Cortes et al. [64] to classify unlabeled lncRNA-protein pairs. When *C* is bigger, that is, the degree of penalty on the misclassified samples is bigger, the computed accuracy is higher on the training set, however, its generalization ability may decrease, that is, the computed accuracy decreases on the test set. On the contrast, smaller *C* can tolerate some misclassified LPI samples on the training set and the generalization ability of the model thus is stronger. Let the misclassified LPIs are denoted as noises, *C*-SVM can be defined by Eq. (21):

21 $$\begin{aligned} \begin{aligned} \min _{{\varvec{w}}, b, {\xi }}&\frac{1}{2} {\varvec{w}}^{T} {\varvec{w}}+C \sum _{i=1}^{l} {\xi }_{i} \\ \text { subject to }\quad&y_{i}\left( {\varvec{w}}^{T} \phi \left( {\varvec{x}}_{i}\right) +b\right) \ge 1-{\xi }_{i}, \\&{\xi }_{i} \ge 0, i=1, \ldots , l \end{aligned} \end{aligned}$$

where $C>0$ is a penalty coefficient of misclassified LPI samples. $\xi _i$ is a slack variable used to measure the degree of misclassification of data, $\phi \left( {\varvec{x}}_{i}\right)$ is used to map ${\varvec{x}}_{i}$ into a higher-dimensional space and *b* denotes a bias. Considering the high dimensional characteristics of vector variable ${\varvec{w}}$, Cortes at al. [64] solve the model (21) based on Eq. (22):

22 $$\begin{aligned} \begin{aligned} \min _{\varvec{\alpha }}&\frac{1}{2} \varvec{\alpha }^{T} Q \varvec{\alpha }-{\varvec{e}}^{T} \varvec{\alpha } \\ \text {subject to } \qquad&{\varvec{y}}^{T} \varvec{\alpha }=0, \\&0 \le \alpha _{i} \le C, \quad i=1, \ldots , l \end{aligned} \end{aligned}$$

where ${\varvec{e}}=[1, \ldots , 1]^{T}$ denotes a vector with all elements of 1, *Q* is an $l \times l$ positive semidefinite matrix where $Q_{i j} = y_{i} y_{j} K\left( {\varvec{x}}_{i}, {\varvec{x}}_{j}\right)$, and $K\left( {\varvec{x}}_{i}, {\varvec{x}}_{j}\right) = \phi \left( {\varvec{x}}_{i}\right) ^{T} \phi \left( {\varvec{x}}_{j}\right)$ denotes a kernel function.

The optimal ${\varvec{w}}$ can be obtained based on the primal-dual relationship by the model Eq. (23):

23 $$\begin{aligned} {\varvec{w}}=\sum _{i=1}^{l} y_{i} \alpha _{i} \phi \left( {\varvec{x}}_{i}\right) \end{aligned}$$

Thus LPI classification function can be denoted by Eq. (24).

24 $$\begin{aligned} {{\mathrm{sgn}}} ({\varvec{w}^T}\phi (\varvec{x}) + b) = {\mathrm{sgn}} (\sum \limits _{i = 1}^l {{y_i}{\alpha _i}K({\varvec{x}_i},\varvec{x}) + b}) \end{aligned}$$

### A hybrid framework

In the above sections, DNN, XGBoost, and *C*-SVM efficiently capture potential LPIs. However, DNNs need to train more parameters [65], XGBoost may lead to an overfitting state when hyperparameters are not appropriately tuned [59], *C*-SVM needs abundant labeled training data [63]. Ensemble learning demonstrates better classification ability compared to one single classifier [26]. To reduce overfitting and obtain optimal prediction performance, we integrate the three classifiers and develop a hybrid framework for LPI identification by Eq. (25):

25 $$\begin{aligned} Score=\alpha C_{DNN}+\beta C_{XGBoost}+\theta C_{SVM} \end{aligned}$$

where $C_{DNN}$, $C_{XGBoost}$, and $C_{C-SVM}$ represent the classification results of an unlabeled lncRNA-protein pair from DNN, XGBoost, and *C*-SVM, respectively. $\alpha$, $\beta$, and $\theta$ indicate the corresponding weights.

## Results

### Evaluation metrics

We use six evaluation metrics to measure the classification ability of our proposed LPI-HyADBS framework. That is, precision, recall, accuracy, F1-score, AUC and AUPR. For the six measurements, higher values indicate better prediction performance. The experiments are repeatedly performed 20 times and the average performance for the 20 experiments is taken as the final results.

### Experimental settings

Pyfeat is applied to extract lncRNA and protein features. The parameters in Pyfeat for lncRNA initial feature acquisition are set as: kGap = 5, kTuple = 3, opti-mumDataset = 1, pseudoKNC = 1, zCurve = 1, gcContent = 1, cumulativeSkew = 1, atgcRatio = 1, monoMono = 1, monoDi = 1, monoTri = 1, diMono = 1, diDi = 1, diTri = 1, triMono = 1, triDi = 1.

The parameters in Pyfeat for protein initial feature acquisition are set as: kGap = 5, kTuple = 3, opti-mumDataset = 1, pseudoKNC = 1, zCurve = 0, gcContent = 0, cumulativeSkew = 0, atgcRatio = 0, monoMono = 1, monoDi = 0, monoTri = 0, diMono = 0, diDi = 0, diTri = 0, triMono = , triDi = 0.

To tune parameters and avoid overfitting, we perform the following experimental settings in DNN: (1) Original settings: an original neural network with one hidden layer is built, where learning rate, epoch, and batch size are originally set to 0.1, 200, and 64, respectively. The number of intermediate layers is selected based on the classification results on dataset 1. (2) Loss function: mean absolute deviation, mean square error, and binary cross-entropy loss [66] are used as loss functions to evaluate the performance of DNN, respectively. Finally, binary cross-entropy loss is selected as loss function because DNN computes better performance using binary cross-entropy loss function. (3) Optimizer: stochastic gradient descent, average stochastic gradient descent, adaptive gradient, and adaptive moment estimation [67] are used as optimizer, respectively. Finally, adaptive moment estimation is selected as optimizer due to the optimal classification ability of DNN. (4) Learning rate, epoch, and batch size: the three parameters are set to corresponding optimal values by grid research. (5) Activation function: LPI classification capability of DNN based on tanh and ReLU is compared and ReLU is selected as activation function in the hidden function where DNN calculates better performance. (6) Dropout: LPI identification accuracy of DNN does not significantly change when dropout is set as 0.2, 0.25, 0.3, and 0.5, therefore, dropout is selected as 0.25 where DNN obtains slightly better performance on dataset 1. (7) Iteration termination: during training, the iteration will be terminated when accuracy is greater than or equal to 0.99 to avoid overfitting.

In SVM, each LPI features are standardized because the selected features based on AdaBoost have multiple dimensions and scales. In addition, SVM is not sensitive to selection of kernel functions on five LPI datasets. Radial basis function (RBF), polynomial function, and sigmoid function are taken as kernel functions to measure LPI classification ability of SVM, respectively. After comparison, SVM with RBF gains slightly better prediction accuracy, therefore, RBF is selected as kernel function.

In XGBoost, parameters are originally set as defaults. Because there are many parameters in XGBoost, the parameters are combined in pairs. And the optimal parameter combination can be obtained by grid search for each group. In the training process, validation set is used to achieve the early stop mechanism of XGBoost and effectively avoid overfitting.

LPI-NRLMF, Capsule-LPI, LPI-CNNCP, LPLNP, and LPI-HyADBS obtain the best performance when they select the optimal parameter combinations by grid search. The optimal parameter combinations for the five methods are shown in Table 2. The parameters in LPI-SKF and LPBNI are set to corresponding values provided by Zhou et al. [23] and Ge et al. [18], respectively.

**Table 2:** Parameter settings

Method	Parameter setting
LPI-NRLMF	Cfix = 5, num_factors = 10, K1 = 5, max_iter = 100
	Lambda_t = 0.625, alpha = 0.1, beta = 0.1
	K2=5, theta = 1.0, lambda_d = 0.625
Capsule-LPI	EPOCH = 30, lr = 0.001, BATCH_SIZE = 100
LPI-CNNCP	Filters1 = 24, kernel_size1 = (49, 10)
	Kernel_size2 = (64, 10), strides2 = (1, 3)
	Strides1 = (1, 1), filters2 = 24
LPI-HyADBS	DNN: Adam(model.parameters(), lr = 0.0001),
	Loss_fn=BCELoss(), batch = 128, epochs = 100
	XGBoost: learning_rate = 0.1, n_estimators = 100
	Objective =“binary:logistic”, max_depth = 6
	*C*-SVM: kernel=“rbf”, gamma = “auto”,
	Probability = True, colsample_btree = 0.8
	$\alpha =0.4$, $\beta = 0.3$, $\theta = 0.3$
LPLNP	Neighbor_num = [6, 23, 100, num of lncRNA-100, 100],
	Regulation = ’regulation2’, alpha = [0.5, 0.3, 0.7, 0.1, 0.9]

Four different 5-fold CVs are implemented to investigate the performance of LPI-HyADBS.

1. 5-fold CV on lncRNAs ($CV_l$): random rows in *Y* are hidden for testing, that is, 80% of lncRNAs are randomly screened as the train set and the remaining are applied to the test set.
2. 5-fold CV on proteins ($CV_p$): random columns in *Y* are hidden for testing, that is, 80% of proteins are randomly screened as the train set and the remaining are applied to the test set.
3. 5-fold CV on lncRNA-protein pairs ($CV_{lp}$): random lncRNA-protein pairs in *Y* are hidden for testing, that is, 80% of lncRNA-protein pairs are randomly screened as the train set and the remaining are applied to the test set.
4. 5-fold CV on independent lncRNAs and independent proteins ($CV_{ind}$) [68]: First, 20% of lncRNAs and 20% of proteins are randomly screened to construct the “node test set”. Second, the remaining nodes, which contain lncRNAs and proteins, are used as the “node train set”. Third, all edges linking a node from the node train set with a node from the node test set are removed. Finally, one classification model is trained only on edges linking two nodes within the node train set to infer edges linking two nodes within the node test set.

The above four CVs correspond to potential LPI identification for (1) new (unknown) lncRNAs without linkages with any protein, (2) new proteins without linkages with any lncRNA, (3) new lncRNA-protein pairs, and (4) the constructed independent lncRNA-independent protein pairs.

More importantly, negative samples (non-LPIs) are randomly screened from unknown lncRNA-protein pairs. The number of negative samples is set to the same as that of positive samples (LPIs).

### Comparison with six state-of-the-art LPI prediction methods

We compare the proposed LPI-HyADBS framework with six classical LPI inference models, that is, LPI-SKF, LPI-NRLMF, Capsule-LPI, LPI-CNNCP, LPLNP, and LPBNI to investigate the classification ability of LPI-HyADBS. LPI-SKF, LPLNP, and LPBNI are three network-based methods, LPI-NRLMF is a logistic matrix factorization-based approach with neighbor regularization, Capsule-LPI and LPI-CNNCP are two deep learning-based models.

**Fig. 3:**
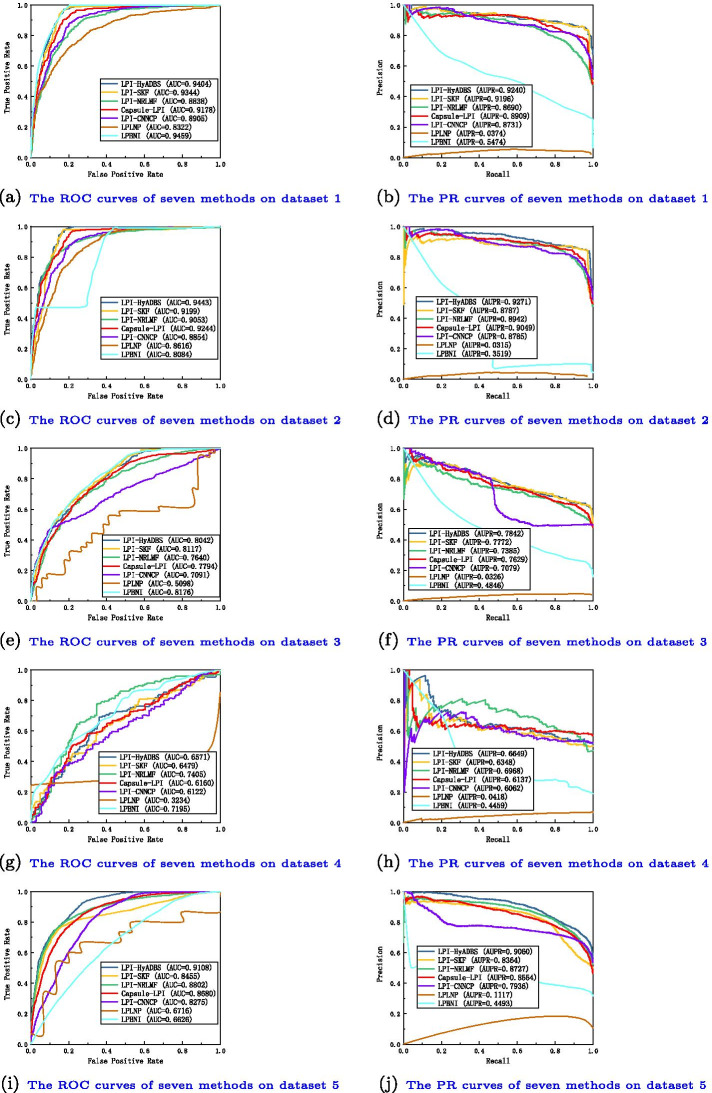
The ROC curves and the PR curves of seven methods under $CV_{l}$

Table I in Additional File 1 show the precision, recall, accuracy, F1-score, AUC and AUPR values obtained from LPI-SKF, LPI-NRLMF, Capsule-LPI, LPI-CNNCP, LPLNP, LPBNI, and LPI-HyADBS on five datasets under $CV_l$. Figure 3 illustrates the ROC and PR curves of the seven LPI prediction methods under $CV_l$. From Table I, we can observe that LPI-HyADBS computes the best average precision, AUC, and AUPR on five datasets under $CV_l$. In particular, LPI-HyADBS computes the best average AUC of 0.8514, better 2.29%, 1.96%, 3.56%, 7.81%, 24.86%, and 7.12% than LPI-SKF, LPI-NRLMF, Capsule-LPI, LPI-CNNCP, LPLNP, and LPBNI, respectively. LPI-HyADBS obtains the highest average AUPR of 0.8412, outperforming 3.79%, 3.21%, 3.24%, 8.25%, 93.94%, and 45.82% compared the above six models, respectively. Although the average F1-score calculated by LPI-HyADBS is lower than one from Capsule-LPI, the difference is very small. For example, Capsule-LPI computes the average F1-score of 0.7570, while LPI-HyADBS obtains the average F1-score of 0.7535, which is only lower 0.46% than Capsule-LPI. Although LPLNP and LPBNI computes better average recall and accuracy than LPI-HyADBS, respectively, LPI-HyADBS markedly outperforms the two methods in terms of average AUC and AUPR. More importantly, AUC and AUPR can more precisely depict the prediction performance of LPI identification techniques compared to the other four evaluation metrics. LPI-HyADBS obtains better AUCs and AUPRs, and can thus accurately find proteins interacting with a new lncRNA.

**Fig. 4:**
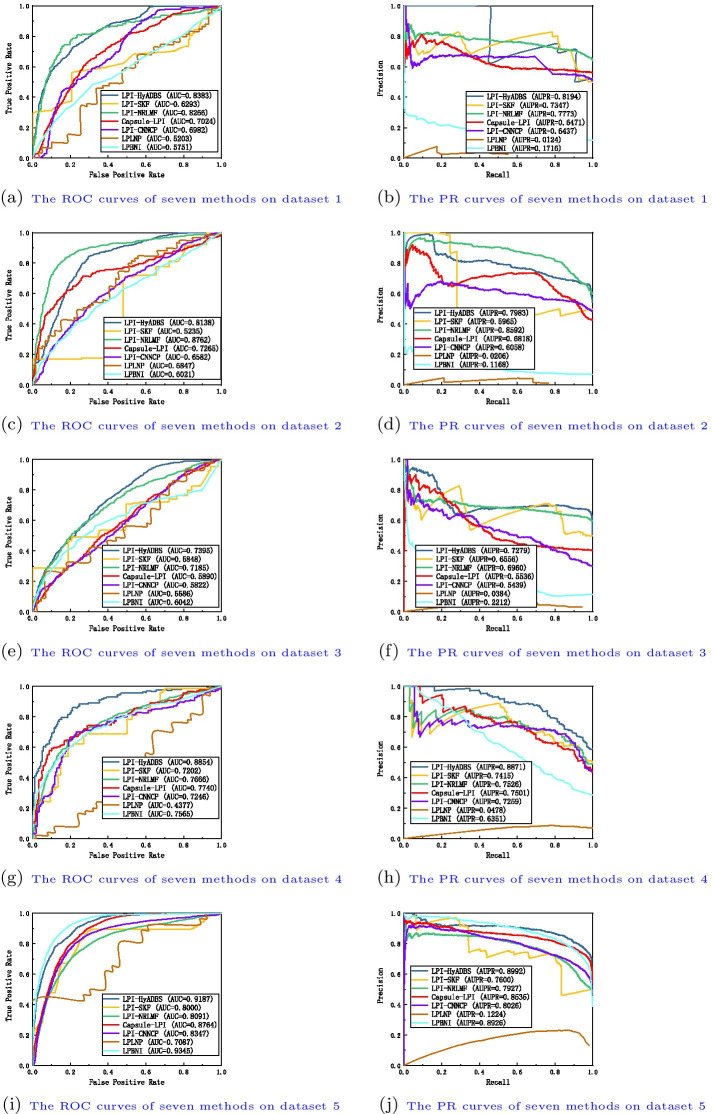
The ROC curves and the PR curves of seven methods under $CV_{p}$

Table II in Additional File 2 illustrates the precision, recall, accuracy, F1-score, AUC and AUPR values calculated by LPI-SKF, LPI-NRLMF, Capsule-LPI, LPI-CNNCP, LPLNP, LPBNI, and LPI-HyADBS on five datasets under $CV_p$. Figure 4 describes the ROC and PR curves of the seven LPI prediction methods under $CV_p$. From Table II, we can find that LPI-HyADBS computes the best average precision, F1-score, AUC, and AUPR. In particular, there are only 59, 84, 27, 35, and 42 proteins on five datasets, respectively. Under $CV_p$, only 80% samples (proteins) are used to train the model on five datasets, respectively. That is, the number of samples is relatively smaller. However, LPI-HyADBS outperforms the other six methods and significantly boosts the performance of LPI prediction. For example, the average AUC computed by LPI-HyADBS exceeds 4.73% and 11.23% than the best and the second-best methods (LPI-NRLMF and Capsule-LPI), respectively. AUPR from LPI-HyADBS is better 6.16% and 15.57% than the best two methods (LPI-NRLMF and LPI-SKF). Although LPBNI computes better accuracy, its calculated AUC and AUPR are obviously smaller than ones from LPI-HyADBS. The results suggest that LPI-HyADBS is a more robust classifier even under relatively smaller samples.

**Fig. 5:**
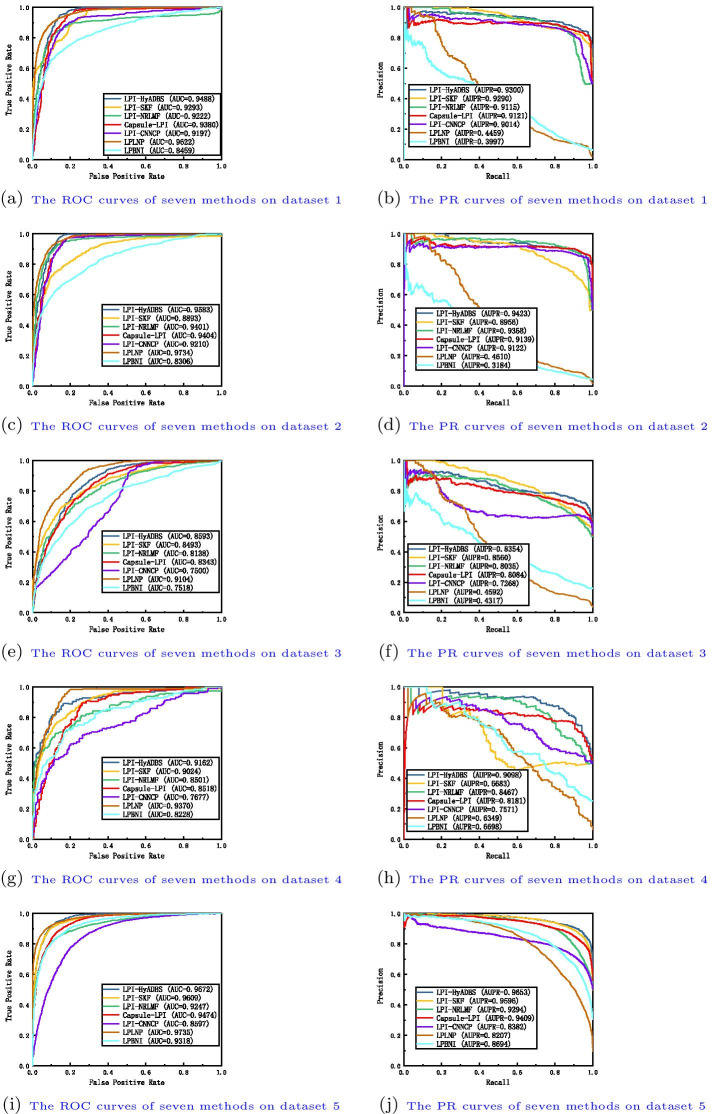
The ROC curves and the PR curves of seven methods under $CV_{lp}$

**Fig. 6:**
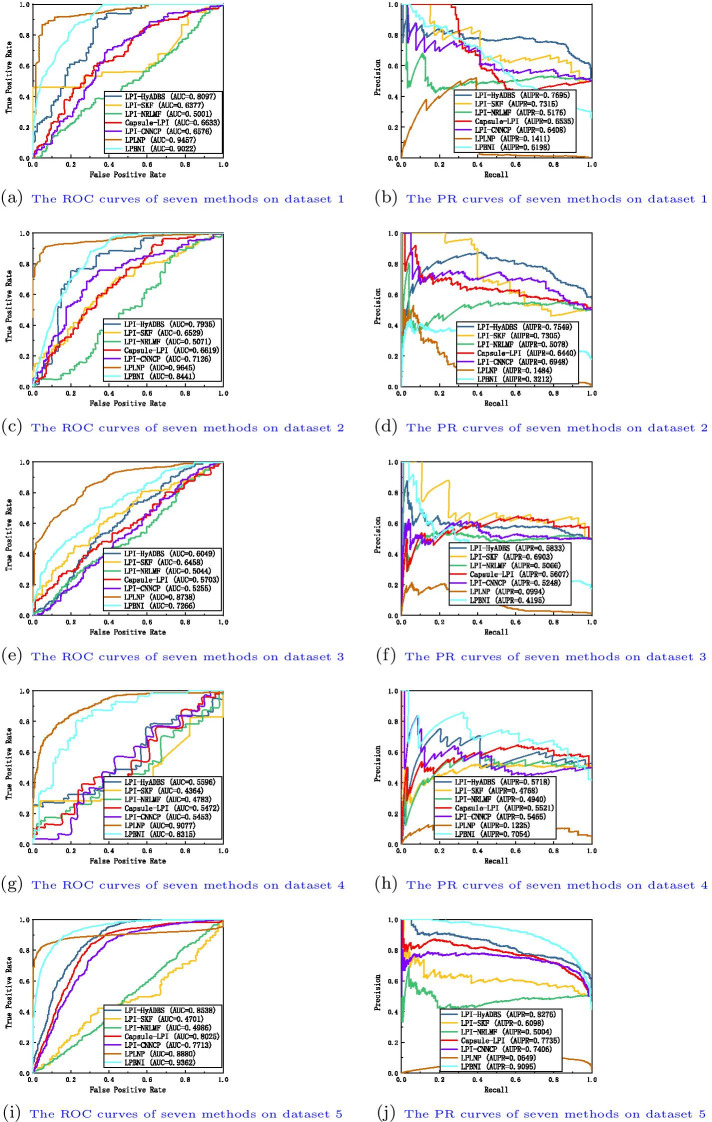
The ROC curves and the PR curves of seven methods under $CV_{ind}$

Table III in Additional File 3 depicts the precision, recall, accuracy, F1-score, AUC and AUPR values achieved from LPI-SKF, LPI-NRLMF, Capsule-LPI, LPI-CNNCP, LPLNP, LPBNI, and LPI-HyADBS on five datasets under $CV_{lp}$. Figure 5 characterizes the ROC and PR curves of the seven LPI prediction methods under $CV_{lp}$. Under $CV_{lp}$, LPI-HyADBS computes the best average performance in terms of precision, recall, F1-score, and AUPR. In particular, LPI-HyADBS calculates the best F1-score on all five datasets. It still obtains the highest average F1-score of 0.8715, outperforming 19.46%, 11.50%, 2.56%, 32.46%, 97.92%, and 54.54% than LPI-SKF, LPI-NRLMF, Capsule-LPI, LPI-CNNCP, LPLNP, and LPBNI, respectively. In addition, LPI-HyADBS calculates the best AUPRs on datasets 1, 2, 4, and 5. The average AUPR is 0.9166, better 5.99%, 3.41%, 4.15%, 9.76%, 38.44%, and 41.33% than the above six approaches, respectively. The results bring out the optimal LPI classification ability of the proposed LPI-HyADBS under $CV_{lp}$.

Table IV in Additional File 4 reveals the precision, recall, accuracy, F1-score, AUC and AUPR values acquired from LPI-SKF, LPI-NRLMF, Capsule-LPI, LPI-CNNCP, LPLNP, LPBNI, and LPI-HyADBS on five datasets under $CV_{ind}$. Figure 6 displays the ROC and PR curves of the seven LPI prediction methods under $CV_{ind}$. Under $CV_{ind}$, the performance of all seven classifiers drastically declines on five datasets. However, LPI-HyADBS achieves better average precision and AUPR than the other six models even under $CV_{ind}$. The average AUPR calculated by LPI-HyADBS is higher 7.64%, 27.97%, 9.22%, 10.25%, 83.56%, and 15.16% than the above six approaches, respectively. Although the AUC, accuracy, and recall values from LPLNP are better than LPI-HyADBS, its precision, F1-score and AUPR values are abnormally behind our method. The performance of LPI-HyADBS is much more stable compared to LPLNP. The results from $CV_{ind}$ again demonstrate the superior LPI identification capability of LPI-HyADBS.

### Performance comparison of single classifiers and hybrid framework

**Fig. 7:**
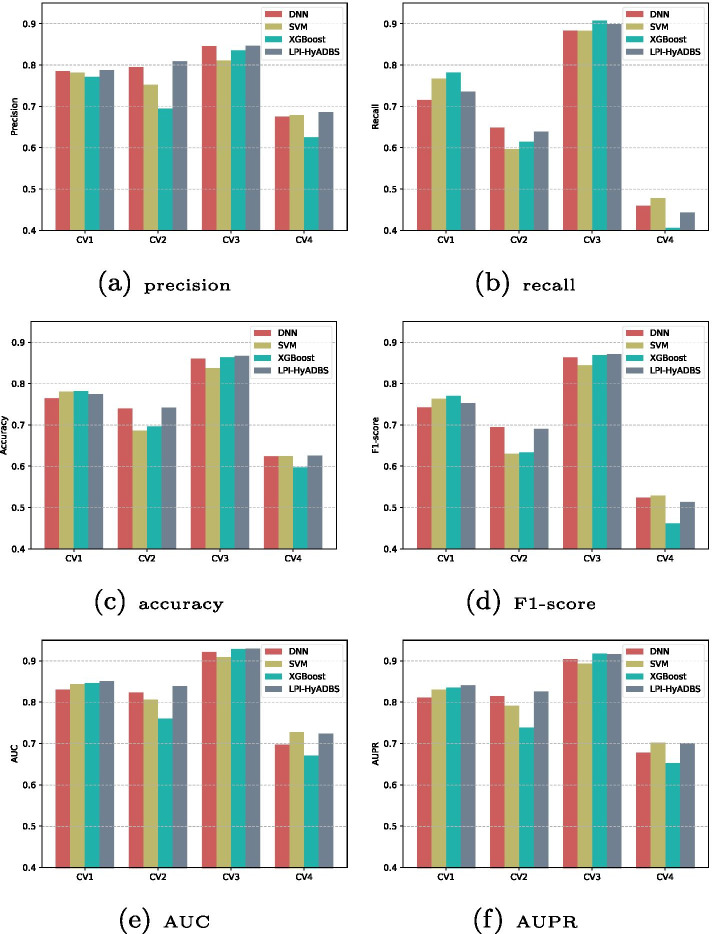
The performance of three single classifiers and a hybrid framework under four cross validations

In this section, each single classifier is compared with the proposed LPI-HyADBS framework to measure the performance of a single classifier with a hybrid method. LPI-HyADBS is a hybrid framework composed of DNN, XGBoost, and *C*-SVM. Figure 7 illustrates the precisions, recalls, accuracies, F1-scores, AUCs, and AUPRs from the three classifiers and LPI-HyADBS. From Fig. 7, we can observe that LPI-HyADBS obtains better precision, F1-score, AUC, and AUPR compared to the other three approaches under all four CVs. In particular, Under $CV_p$ and $CV_{ind}$, LPI-HyADBS is significantly superior to the other three methods. The results suggest that LPI-HyADBS, ensemble of DNN, XGBoost, and *C*-SVM, can improve LPI prediction performance.

### Performance comparison of single classifiers based on deep learning

**Fig. 8:**
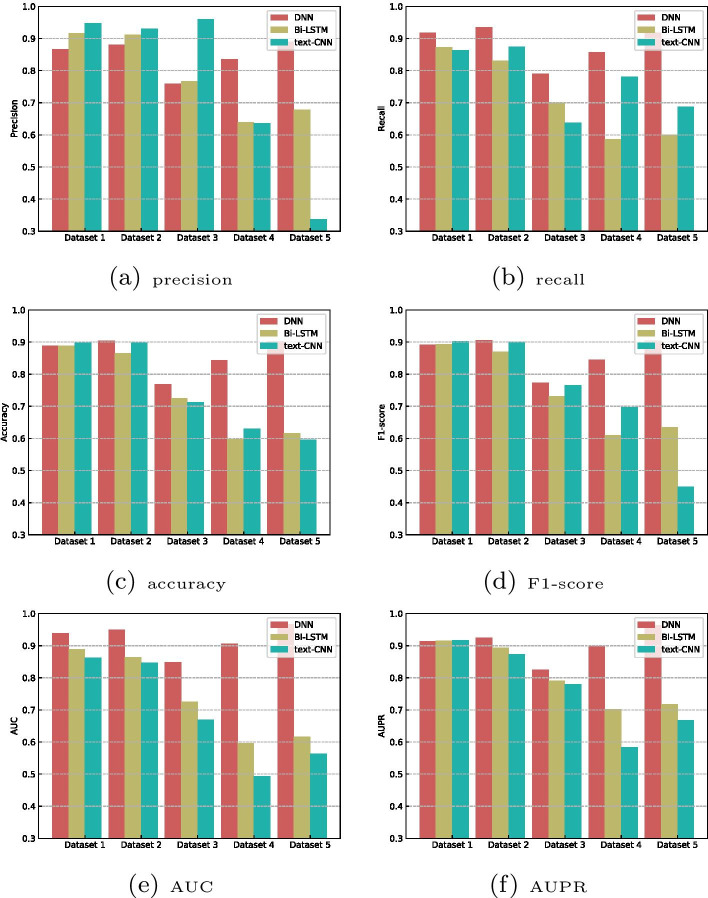
Performance comparison of single classifiers based on deep learning

In the proposed LPI-HyADBS framework, DNN, as one single classifier based on deep learning, gains better LPI prediction performance. To investigate the performance of the other deep learning-based models on LPI discovery, we compare DNN with two classical deep learning-based methods, that is, Text-attentional CNN (Text-CNN) [69] and Bi-LSTM [40]. Text-CNN [69] focused on extracting text-related features from image components and effectively detected highly challenging text patterns. Bi-LSTM [40] revealed underlying long range dependencies between RNA binding sequences and structure motifs from RNA sequences. The two methods computed better performance on corresponding applications. Figure 8 describes the comparison results of DNN with Text-CNN and Bi-LSTM. From Fig. 8, we can observe that DNN significantly outperforms Text-CNN and Bi-LSTM in terms of recalls, accuracies, F1-scores, AUCs and AUPRs on five datasets in the vast majority of cases. The results demonstrate that DNN may be more appropriate for underlying LPI detection.

### Case study

In this section, we investigate the application of the proposed LPI-HyADBS method.

#### Finding possible proteins for a new lncRNA

RNase MRP RNA is an abundant and essential noncoding RNA. The functions of RNase MRP RNA are still incompletely understood in humans. Mutations on RNase MRP RNA genes may cause a recessively inherited developmental disorder, that is, cartilage-hair hypoplasia [70]. Cartilage-hair hypoplasia is highly human pleiotropic. It has dense associations with defective cellular immunity and short stature. More importantly, it may cause multiple cancers [71].

In human datasets 1–3, RNase MRP RNA (its name is NONHSAT130962, n5543, NONHSAT130962, respectively) interacts with 3, 13, and 10 proteins, respectively. To infer possible proteins linking with RNase MRP RNA, all its associated proteins are hidden and it is regarded as a new lncRNA. LPI-HyADBS together with the other six comparison methods are applied to infer the relevances between RNase MRP RNA and proteins. The predicted top 5 proteins linking with RNase MRP RNA are shown in Table 3. In dataset 1, P35367, O00425, Q9Y6M1, and Q9NZI8 are predicted to have high association probabilities with RNase MRP RNA. P35637 is known to interact with RNase MRP RNA in dataset 2, O00425 and Q9NZI8 have been confirmed to associate with RNase MRP RNA in dataset 3, and Q9Y6M1 is reported association information with RNase MRP RNA in datasets 2 and 3. Although interactions between Q9NZI8 and and RNase MRP RNA, and between P35367 and RNase MRP RNA are unknown in datasets 2 and 3, respectively, they have been validated in datasets 3 and 2, respectively. In summary, the predicted top 5 proteins interacting with RNase MRP RNA in one human dataset can be confirmed in the other two datasets.

**Table 3:** The predicted top 5 proteins interacting with RNase MRP RNA

Dataset	Proteins	Confirmed	LPI-HyADBS	LPI-SKF	NRLMF	Capsule	CNNCP	LPLNP	LPBNI
Dataset 1	Q15717	YES	1	1	1	1	43	3	4
	P35637	NO	2	4	5	3	47	6	22
	O00425	NO	3	7	2	2	8	4	1
	Q9Y6M1	NO	4	6	3	5	11	7	40
	Q9NZI8	NO	5	9	4	9	46	12	15
Dataset 2	Q15717	YES	1	9	1	3	71	11	6
	P35637	YES	2	1	3	4	39	9	54
	Q9NZI8	NO	3	12	4	10	47	6	12
	Q9Y6M1	YES	4	14	2	9	78	54	7
	P31483	YES	5	10	9	7	84	2	9
Dataset 3	Q9NUL5	YES	1	5	1	1	26	7	6
	Q9Y6M1	YES	2	6	3	4	2	2	11
	Q9NZI8	YES	3	10	5	3	16	6	5
	P35637	NO	4	11	4	5	6	1	4
	O00425	YES	5	7	2	2	25	9	18

#### Finding possible lncRNAs for a new protein

P35637 involves in multiple cellular processes. The processes include transcription regulation, DNA repair and damage response, RNA splicing and transport [72]. In neuronal cells, P35367 plays crucial roles in RNA transport, mRNA stability, dendritic spine formation and stability, and synaptic homeostasis [46].

P35637 may interact with 935, 885, and 990 lncRNAs on datasets 1–3, respectively. We hide all linkage data for P35367 and utilize the proposed LPI-HyADBS framework to infer lncRNAs related to P35367. The predicted top 5 relevant lncRNAs on three human datasets are shown in Table 4. In dataset 2, interaction between hTR and P35367 is known in dataset 3; interaction between 7SL and P35367 has been confirmed in datasets 1 and 3. However, interactions between P35367 and two lncRNAs (RPI001_1039837 and RN7SK) can not been validated. RN7SK is a small nuclear RNA involved in cellular senescence [73] and neuronal differentiation [74], it regulates macrophage polarization and innate immune responses [75]. The interaction between RN7SK and P35367 is ranked as 4 and 2 by LPI-HyADBS and LPI-NRLMF, respectively. We infer RN7SK may interact with P35367 and need further validation.

**Table 4:** The predicted top 5 lncRNAs interacting with P35637

Dataset	lncRNAs	Confirmed	LPI-HyADBS	LPI-SKF	NRLMF	Capsule	CNNCP	LPLNP	LPBNI
Dataset 1	RPI001_966611	YES	1	206	1	198	858	6	19
	RPI001_1030838	YES	2	241	50	220	652	52	201
	RPI001_171640	YES	3	111	97	106	829	65	2
	RPI001_1039837	NO	4	897	6	232	812	369	55
	CTD-2350C19.1	YES	5	211	9	182	920	15	74
Dataset 2	n343060	YES	1	111	1	845	360	2	9
	hTR	NO	2	802	457	199	253	8	13
	RMRP	YES	3	119	4	581	843	55	289
	RN7SK	NO	4	286	2	678	390	72	166
	7SL	NO	5	311	45	177	385	117	11
Dataset 3	NONHSAT006903	YES	1	144	12	146	449	39	162
	PTENP1	YES	2	26	5	60	471	18	33
	RPI001_112304	YES	3	169	15	407	42	196	27
	RPI001_634699	YES	4	224	32	61	190	12	61
	RPI001_111205	YES	5	119	50	370	414	81	94

#### Finding possible LPIs based on observed LPIs

**Fig. 9:**
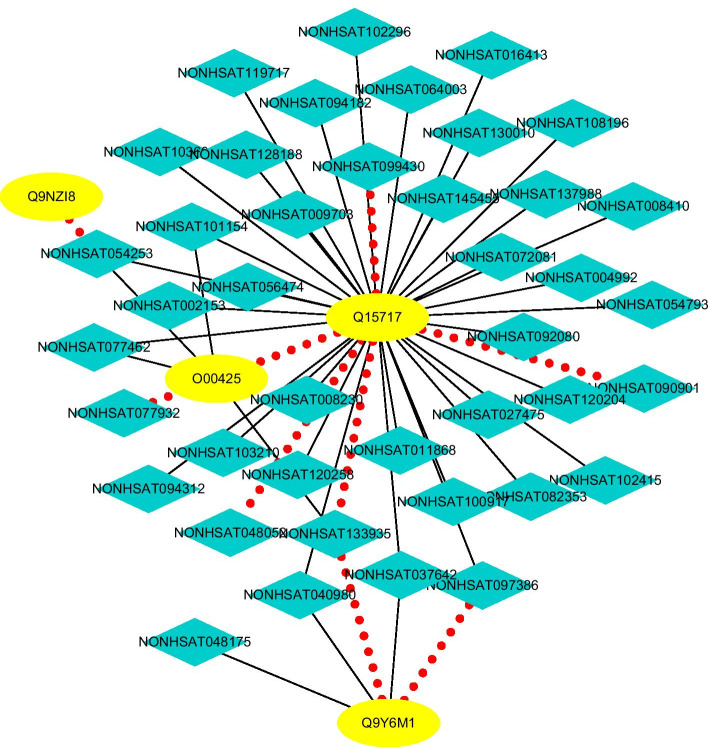
The predicted top 50 LPIs on Dataset 1

**Fig. 10:**
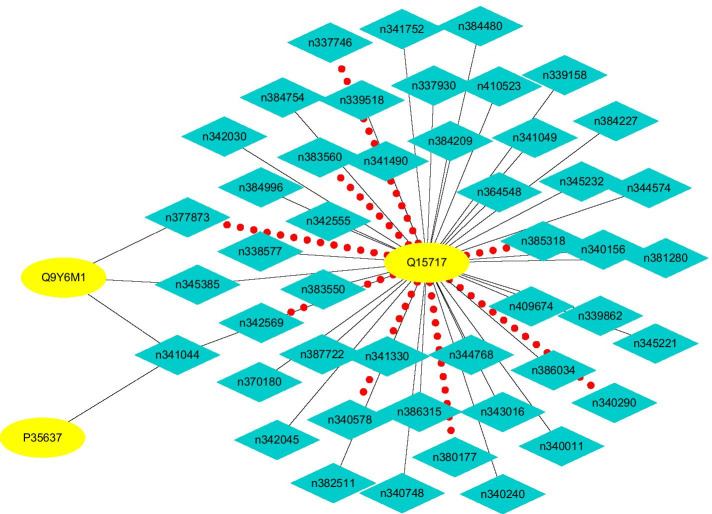
The predicted top 50 LPIs on Dataset 2

**Fig. 11:**
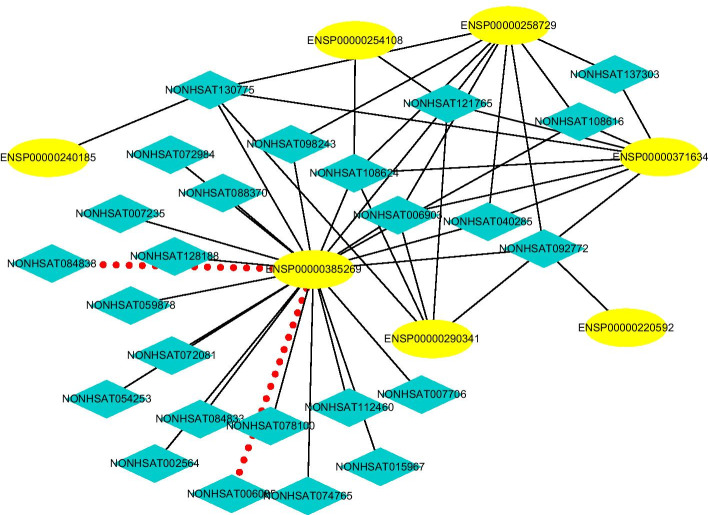
The predicted top 50 LPIs on Dataset 3

**Fig. 12:**
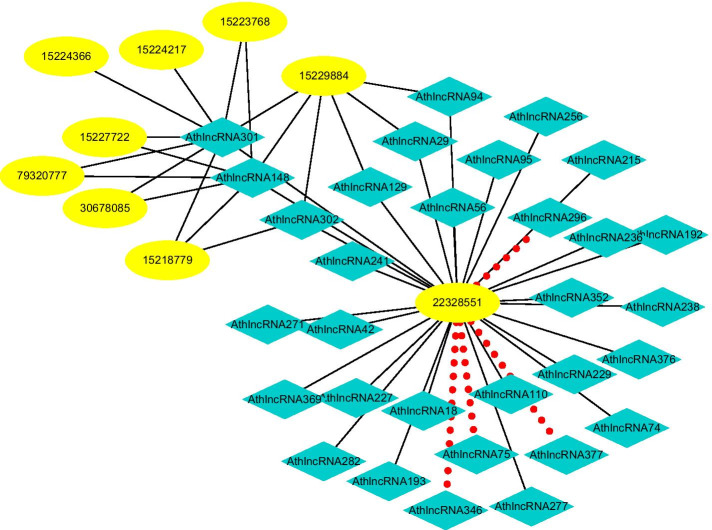
The predicted top 50 LPIs on Dataset 4

**Fig. 13:**
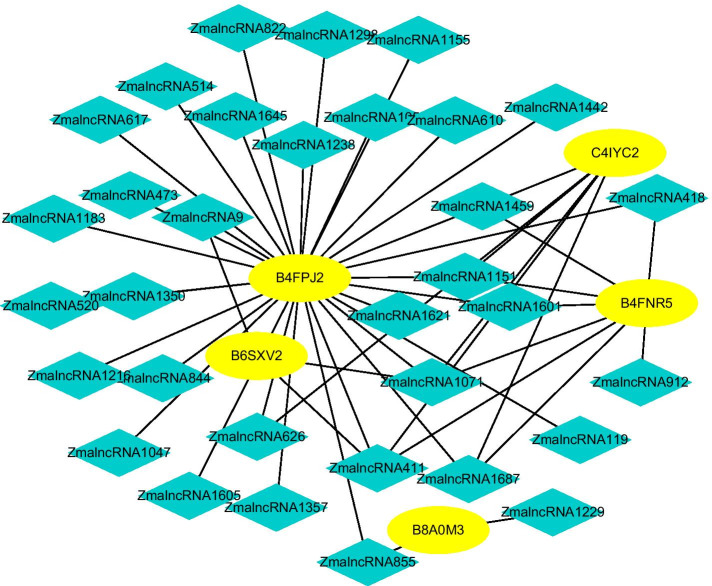
The predicted top 50 LPIs on Dataset 5

We score each lncRNA-protein pair on datasets 1–5. Figures 9, 10, 11, 12 and 13 illustrate the discovered top 50 lncRNA-protein pairs with the highest interaction probabilities. In the figures, black solid lines and red dotted lines represent known and unknown LPIs obtained from LPI-HyADBS, respectively. Deep sky blue diamonds represent lncRNAs. Yellow ellipses denote proteins.

On five datasets, there are separately 55,165, 74,340, 26,730, 3815, and 71,568 lncRNA-protein pairs, respectively. Unknown lncRNA-protein pairs between NONH-SAT048052(RP11-561C5.4) and Q15717, n383560(ZNF667-AS1) and Q15717, NONHSAT006085(RPI0-01_1004095) and Q9NUL5, AthlncRNA296(TCONS_0004-9605) and F4JLJ3, and ZmalncRNA1655 and B8A0M3 show the highest interaction probabilities, respectively. The five pairs are rank as 3, 10, 22, 15, and 1619 among all lncRNA-protein pairs, respectively.

ZNF667-AS1 play important roles in aberrant methylation and downregulation [76]. The lncRNA can inhibit inflammatory response [77], proliferation of cervical cancer [78], and progression of colorectal cancer [79], reduce tumor invasion and metastasis in cervical cancer [80], and promote recovery of spinal cord injury [77]. Q15717 has close relevance with embryonic stem cells differentiation. The protein interacts with ZNF385A to control nuclear export induced by CDKN2A and mediate in part the CDKN2A anti-proliferative activity. Both ZNF667-AS1 and Q15717 densely link with the inhibition of proliferation, and interaction between ZNF667-AS1 and Q15717 need experimental validation.

## Discussion and conclusion

lncRNAs have dense connections with multiple physiological and pathological processes by interacting with proteins. In this manuscript, we develop an LPI inference framework combining an LPI feature selection algorithm based on AdaBoost and an ensemble learning model composed of DNN, XGBoost, and *C*-SVM. To observe the performance of the proposed LPI-HyADBS framework, we compare it with six representative LPI prediction approaches on five datasets under four different CVs. The six methods are LPI-SKF, LPI-NRLMF, Capsule-LPI, LPI-CNNCP, LPLNP, and LPBNI. LPI-SKF, LPLNP, and LPBNI are three representative network-based LPI prediction models. LPI-NRLMF is a classical matrix factorization-based LPI identification approach. Capsule-LPI and LPI-CNNCP are two state-of-the-art deep learning-based LPI classification models.

Under all four different CVs, LPI-HyADBS achieves better prediction performance, significantly outperforming the other six approaches. The results demonstrate the strong classification ability of LPI-HyADBS. In particular, under $CV_{p}$, only smaller samples are applied to train the model in each round. However, LPI-HyADBS still computes the best performance, showing its robustness under small samples. More importantly, $CV_{ind}$ is conducted on independent lncRNAs and independent proteins. Under $CV_{ind}$, all edges connecting a node from the node train set with another node from the node test set are removed. And seven LPI identification approaches are trained only on edges connecting two nodes within the node train set to infer interactions between two nodes within the node test set. $CV_{ind}$ reduces the overfitting problem of the classification models. LPI-HyADBS obtains better performance than the other six approaches even under $CV_{ind}$. The results again show the robustness of LPI-HyADBS.

Capsule-LPI and LPI-CNNCP are two deep learning-based LPI prediction algorithms. From Tables I–IV in the Supplementary Materials and Figs. 3, 4, 5 and 6, we can find that LPI-HyADBS outperforms the two deep learning-based LPI inference models. More importantly, LPI-HyADBS integrates DNN, XGBoost, and *C*-SVM. Figure 7 illustrates that LPI-HyADBS improves LPI prediction ability compared to the three basic classifiers. The results indicate that deep ensemble-based models may more accurately find possible interplays between lncRNAs and proteins. In addition, LPI-HyADBS calculates the best performance on datasets 1, 2, 4, and 5. On dataset 3, LPI-HyADBS achieves relatively lower performance. It may be resulted in by different structures of data.

LPI-HyADBS can precisely predict the relevances between lncRNAs and proteins. It may be attributed to the following advantages. First, LPI-HyADBS fuses various biological characteristics for LPI prediction. Second, the feature selection algorithm based on AdaBoost selects the informative LPI features. Finally, an ensemble learning framework, composed of DNN, XGBoost, and *C*-SVM, integrates the merits of the three basic classifiers and can more effectively classify unlabeled lncRNA-protein pairs.

Although LPI-HyADBS computes the best performance on three human datasets and two plant datasets, considering other species more relative to human may more accurately evaluate LPI prediction models. Therefore, in the future, we will integrate existing data sources and construct LPI datasets for other species closer to human.

## Supplementary information

**Additional file 1**: ** Table SI**. The performance of seven LPI prediction methods on CV_l_, the precision, recall, accuracy, F1-score, AUC and AUPR values obtained from LPI-SKF, LPI-NRLMF, Capsule-LPI, LPI-CNNCP, {LPLNP, LPBNI,} and LPI-HyADBS on five datasets under CV_l_.

**Additional file 2**: ** Table SII** The performance of seven LPI prediction methods on CV_p_, the precision, recall, accuracy, F1-score, AUC and AUPR values obtained from LPI-SKF, LPI-NRLMF, Capsule-LPI, LPI-CNNCP, LPLNP, LPBNI, and LPI-HyADBS on five datasets under CV_p_.

**Additional file 3**:** Table SIII**. The performance of seven LPI prediction methods on CV_lp_, the precision, recall, accuracy, F1-score, AUC and AUPR values obtained from LPI-SKF, LPI-NRLMF, Capsule-LPI, LPI-CNNCP, LPLNP, LPBNI, and LPI-HyADBS on five datasets under CV_lp_.

**Additional file 4**:** Table SIV**. The performance of seven LPI prediction methods on CV_ind_, the precision, recall, accuracy, F1-score, AUC and AUPR values obtained from LPI-SKF, LPI-NRLMF, Capsule-LPI, LPI-CNNCP, LPLNP, LPBNI, and LPI-HyADBS on five datasets under CV_ind_.

## Data Availability

Source codes and datasets are freely available for download at https://github.com/plhhnu/LPI-HyADBS.

## Abbreviations

LPI-HyADBS: A hybrid framework integrating feature selection based on AdaBoost, and classification models including DNN, XGBoost, and SVM used to predict LPIs
lncRNA: Long noncoding RNA
LPI: lncRNA-protein interaction
CVs: Cross validations
AdaBoost: Adaptive boosting
XGBoost: eXtreme gradient boosting
SVM: Support vector machine
*C*-SVM: SVM with a penalty coefficient of misclassification
DNN: Deep neural network
CNN: Convolutional neural network
Text-CNN: Text-attentional CNN
Bi-LSTM: Bidirectional long short term memory network
