# Supplementary material for: LPI-HyADBS: a hybrid framework for lncRNA-protein interaction prediction integrating feature selection and classification
Source: BMC Bioinformatics. 2021 Nov 26;22:568. doi: 10.1186/s12859-021-04485-x (PMC8620196; doi:10.1186/s12859-021-04485-x)
Supplement: Supplementary file 1 — Additional file 1: Table SI. The performance of seven LPI prediction methods on CVl, the precision, recall, accuracy, F1-score, AUC and AUPR values obtained from LPI-SKF, LPI-NRLMF, Capsule-LPI, LPI-CNNCP, {LPLNP, LPBNI,} and LPI-HyADBS on five datasets under CVl. [file 12859_2021_4485_MOESM1_ESM.pdf]

Table I The performance of seven LPI prediction methods on CV_l

Metric	Dataset	LPI-SKF	LPI-NRLMF	Capsule-LPI	LPI-CNNCP	LPLNP	LPBNI	LPI-HyADBS
Precision	Dataset 1	**0.8757±0.0086**	0.7932±0.0142	0.8256±0.0150	0.6775±0.1721	0.0531±0.0011	0.5508±0.0024	0.8588±0.0115
	Dataset 2	0.8627±0.0223	0.8119±0.0147	0.8473±0.0139	0.7071±0.2742	0.0435±0.0028	0.4858±0.0328	**0.8697±0.0110**
	Dataset 3	**0.7298±0.0153**	0.6970±0.0140	0.6837±0.0194	0.5731±0.2985	0.0457±0.0026	0.5067±0.0159	0.7142±0.0155
	Dataset 4	0.6108±0.0249	**0.6644±0.0570**	0.5853±0.0846	0.3796±0.3097	0.0628±0.0090	0.4642±0.0984	0.6610±0.0627
	Dataset 5	0.7517±0.0098	0.7830±0.0092	0.8018±0.0145	0.7174±0.0665	0.1647±0.0116	0.3861±0.0535	**0.8335±0.0108**
	Ave.	0.7661	0.7499	0.7487	0.6109	0.0740	0.4787	**0.7874**
Recall	Dataset 1	0.5932±0.0156	0.7216±0.0173	**0.9085±0.0248**	0.8805±0.2297	0.6120±0.0351	0.4315±0.0138	0.8997±0.0213
	Dataset 2	0.5212±0.0107	0.7442±0.0136	0.8924±0.0252	0.6920±0.3275	0.5922±0.1227	0.3805±0.0441	**0.8953±0.0248**
	Dataset 3	0.6226±0.0058	0.6284±0.0117	0.7488±0.0377	0.5390±0.4262	**0.8669±0.0833**	0.3557±0.0236	0.6930±0.0355
	Dataset 4	0.6056±0.0280	0.6032±0.0260	0.5528±0.1120	0.4443±0.4541	**0.9780±0.0307**	0.2805±0.0129	0.3995±0.0818
	Dataset 5	0.6727±0.0037	0.7425±0.0086	0.7588±0.0225	0.7674±0.2291	**0.8356±0.0162**	0.8143±0.0357	0.7894±0.0164
	Ave.	0.6030	0.6879	0.7722	0.6646	**0.7769**	0.4525	0.7354
Accuracy	Dataset 1	0.7254±0.0032	0.7396±0.0100	0.8581±0.0110	0.6601±0.1527	0.8501±0.0051	**0.9402±0.0154**	0.8759±0.0127
	Dataset 2	0.7065±0.0081	0.7596±0.0113	0.8657±0.0134	0.6887±0.1605	0.8800±0.0236	**0.9533±0.0117**	0.8805±0.0121
	Dataset 3	0.6544±0.0092	0.6466±0.0084	0.7010±0.0144	0.5593±0.0768	0.3522±0.0703	**0.8432±0.0036**	0.7075±0.0131
	Dataset 4	0.5727±0.0196	0.6319±0.0178	0.5795±0.0472	0.5123±0.0431	0.0934±0.0384	**0.8027±0.0099**	0.5953±0.0352
	Dataset 5	0.6726±0.0036	0.7366±0.0057	0.7858±0.0089	0.7177±0.0640	0.6385±0.0066	0.5320±0.0312	**0.8162±0.0051**
	Ave.	0.6663	0.7028	0.7580	0.6276	0.5628	**0.8143**	0.7751
F1-score	Dataset 1	0.6298±0.0070	0.7556±0.0140	0.8648±0.0109	0.7163±0.1328	0.0978±0.0018	0.4839±0.0841	**0.8787±0.0131**
	Dataset 2	0.5828±0.0117	0.7765±0.0117	0.8690±0.0134	0.6479±0.2455	0.0808±0.0041	0.4267±0.0133	**0.8821±0.0131**
	Dataset 3	0.5950±0.0086	0.6609±0.0118	**0.7141±0.0188**	0.4374±0.3018	0.0868±0.0051	0.4180±0.0143	0.7028±0.0181
	Dataset 4	0.5401±0.0232	**0.6318±0.0380**	0.5579±0.0692	0.3315±0.3041	0.1179±0.0157	0.3497±0.0984	0.4926±0.0668
	Dataset 5	0.6345±0.0041	0.7622±0.0074	0.7794±0.0128	0.7079±0.1555	0.2750±0.0164	0.5238±0.0658	**0.8112±0.0066**
	Ave.	0.5964	0.7174	**0.7570**	0.5682	0.1317	0.4404	0.7535
AUC	Dataset 1	0.9344±0.0073	0.8838±0.0095	0.9178±0.0088	0.8905±0.0985	0.8322±0.0047	**0.9459±0.0054**	0.9404±0.0083
	Dataset 2	0.9199±0.0149	0.9053±0.0094	0.9244±0.0105	0.8854±0.1481	0.8616±0.0070	0.8084±0.0069	**0.9443±0.0064**
	Dataset 3	0.8117±0.0159	0.7640±0.0122	0.7794±0.0154	0.7091±0.1068	0.5098±0.0167	**0.8176±0.0074**	0.8042±0.0124
	Dataset 4	0.6479±0.0379	**0.7405±0.0286**	0.6160±0.0519	0.6122±0.0436	0.3234±0.0189	0.7195±0.0195	0.6571±0.0432
	Dataset 5	0.8455±0.0076	0.8802±0.0065	0.8680±0.0089	0.8275±0.0258	0.6716±0.0047	0.6626±0.0116	**0.9108±0.0042**
	Ave.	0.8319	0.8347	0.8211	0.7849	0.6397	0.7908	**0.8514**
AUPR	Dataset 1	0.9196±0.0092	0.8690±0.0168	0.8909±0.0138	0.8731±0.0942	0.0374±0.0008	0.5474±0.0110	**0.9240±0.0119**
	Dataset 2	0.8787±0.0260	0.8942±0.0131	0.9049±0.0155	0.8785±0.1182	0.0315±0.0015	0.3519±0.0674	**0.9271±0.0106**
	Dataset 3	0.7772±0.0198	0.7385±0.0190	0.7629±0.0180	0.7079±0.0977	0.0326±0.0016	0.4846±0.0382	**0.7842±0.0158**
	Dataset 4	0.6348±0.0340	**0.6968±0.0619**	0.6137±0.0700	0.6062±0.0647	0.0418±0.0060	0.4459±0.0541	0.6649±0.0430
	Dataset 5	0.8364±0.0170	0.8727±0.0093	0.8554±0.0130	0.7936±0.0327	0.1117±0.0092	0.4493±0.0845	**0.9060±0.0034**
	Ave.	0.8093	0.8142	0.8055	0.7718	0.0510	0.4558	**0.8412**
