# Supplementary material for: LPI-HyADBS: a hybrid framework for lncRNA-protein interaction prediction integrating feature selection and classification
Source: BMC Bioinformatics. 2021 Nov 26;22:568. doi: 10.1186/s12859-021-04485-x (PMC8620196; doi:10.1186/s12859-021-04485-x)
Supplement: Supplementary file 2 — Additional file 2: Table SII The performance of seven LPI prediction methods on CVp, the precision, recall, accuracy, F1-score, AUC and AUPR values obtained from LPI-SKF, LPI-NRLMF, Capsule-LPI, LPI-CNNCP, LPLNP, LPBNI, and LPI-HyADBS on five datasets under CVp. [file 12859_2021_4485_MOESM2_ESM.pdf]

Table II The performance of seven LPI prediction methods on CV_p

Metric	Dataset	LPI-SKF	LPI-NRLMF	Capsule-LPI	LPI-CNNCP	LPLNP	LPBNI	LPI-HyADBS
Precision	Dataset 1	0.7009±0.1208	0.6950±0.1539	0.5925±0.2244	0.4521±0.3563	0.0351±0.0363	0.2509±0.1058	**0.8352±0.0837**
	Dataset 2	0.6138±0.1316	0.7470±0.0920	0.6584±0.1243	0.3336±0.2712	0.0459±0.0130	0.1386±0.1542	**0.8350±0.0958**
	Dataset 3	0.6639±0.1119	0.6355±0.1261	0.5462±0.1292	0.3374±0.2832	0.0617±0.0238	0.3060±0.1984	**0.7075±0.0823**
	Dataset 4	0.7261±0.0412	0.6942±0.0625	0.6990±0.0555	0.4245±0.3211	0.0702±0.0234	0.6530±0.0845	**0.8212±0.0323**
	Dataset 5	0.7264±0.1465	0.7211±0.0428	0.8171±0.0558	0.7194±0.0786	0.1815±0.0475	**0.8711±0.0744**	0.8459±0.0210
	Ave.	0.6862	0.6985	0.6626	0.4534	0.0789	0.4439	**0.8090**
Recall	Dataset 1	0.5415±0.0702	**0.6959±0.0757**	0.5981±0.2899	0.3355±0.3927	0.3665±0.2243	0.1135±0.1145	0.5263±0.1930
	Dataset 2	0.4114±0.0551	**0.7472±0.0438**	0.6265±0.2756	0.5149±0.4482	0.4873±0.2587	0.2794±0.0198	0.4197±0.2144
	Dataset 3	0.4982±0.0746	**0.6388±0.0583**	0.5494±0.2249	0.5271±0.4560	0.5459±0.1032	0.1790±0.0764	0.6222±0.1281
	Dataset 4	0.5402±0.0415	0.6426±0.0385	0.7577±0.1060	0.5069±0.4500	**0.8424±0.0763**	0.4403±0.0186	0.8038±0.0906
	Dataset 5	0.5811±0.0589	0.6899±0.0332	0.7436±0.0878	0.8279±0.2136	**0.8469±0.0608**	0.6803±0.0244	0.8240±0.0579
	Ave.	0.5145	**0.6828**	0.6550	0.5424	0.6178	0.3385	0.6392
Accuracy	Dataset 1	0.5867±0.0757	0.6990±0.0356	0.6610±0.1300	0.5163±0.1325	0.7698±0.1746	**0.8552±0.0028**	0.7144±0.0946
	Dataset 2	0.5220±0.0482	0.7309±0.0298	0.6734±0.1427	0.5358±0.1152	**0.9045±0.0470**	0.8292±0.0084	0.6723±0.1024
	Dataset 3	0.5584±0.0777	0.6225±0.0364	0.5684±0.0982	0.4605±0.1352	0.6852±0.0890	**0.8602±0.0053**	0.6762±0.0657
	Dataset 4	0.6202±0.0332	0.6499±0.0264	0.7118±0.0487	0.5452±0.0798	0.3039±0.1129	0.7732±0.0018	**0.8144±0.0452**
	Dataset 5	0.6636±0.0644	0.6725±0.0158	0.7862±0.0319	0.7347±0.0589	0.6823±0.0139	**0.8373±0.0056**	0.8362±0.0193
	Ave.	0.5902	0.6749	0.6801	0.5585	0.6691	**0.8310**	0.7427
F1-score	Dataset 1	0.5399±0.0745	**0.6902±0.1266**	0.5451±0.2480	0.2651±0.2723	0.0549±0.0529	0.1563±0.1484	0.6281±0.1662
	Dataset 2	0.4092±0.0634	**0.7451±0.0652**	0.6094±0.1877	0.3838±0.3185	0.0816±0.0239	0.1853±0.2153	0.5310±0.2040
	Dataset 3	0.4929±0.0804	0.6337±0.0913	0.5193±0.1467	0.3407±0.2855	0.1098±0.0391	0.2259±0.1655	**0.6525±0.0829**
	Dataset 4	0.5468±0.0408	0.6670±0.0484	0.7208±0.0490	0.3930±0.3138	0.1287±0.0393	0.5260±0.0258	**0.8101±0.0543**
	Dataset 5	0.5908±0.0734	0.7051±0.0376	0.7734±0.0424	0.7408±0.1148	0.2957±0.0614	0.7640±0.0394	**0.8331±0.0259**
	Ave.	0.5159	0.6882	0.6336	0.4246	0.1341	0.3715	**0.6910**
AUC	Dataset 1	0.6293±0.1142	0.8266±0.0724	0.7024±0.1643	0.6982±0.1422	0.5203±0.0957	0.5751±0.0365	**0.8383±0.0822**
	Dataset 2	0.5235±0.0899	**0.8762±0.0321**	0.7265±0.1509	0.6582±0.1138	0.6847±0.0853	0.6021±0.0311	0.8138±0.0990
	Dataset 3	0.5848±0.1577	0.7185±0.0627	0.5890±0.1249	0.5822±0.1115	0.5586±0.1069	0.6042±0.0391	**0.7395±0.0789**
	Dataset 4	0.7202±0.0571	0.7666±0.0408	0.7740±0.0552	0.7246±0.0670	0.4377±0.0594	0.7565±0.0489	**0.8854±0.0416**
	Dataset 5	0.8000±0.1136	0.8091±0.0223	0.8764±0.0270	0.8347±0.0403	0.7087±0.0188	**0.9345±0.0136**	0.9187±0.0132
	Ave.	0.6516	0.7994	0.7336	0.6996	0.5820	0.6945	**0.8391**
AUPR	Dataset 1	0.7347±0.1155	0.7773±0.1660	0.6471±0.1943	0.6437±0.1798	0.0124±0.0116	0.1716±0.1451	**0.8194±0.0891**
	Dataset 2	0.5965±0.1215	**0.8592±0.0739**	0.6818±0.1210	0.6058±0.1250	0.0206±0.0089	0.1168±0.0489	0.7983±0.0975
	Dataset 3	0.6556±0.1277	0.6960±0.1457	0.5536±0.1315	0.5439±0.1800	0.0384±0.0175	0.2212±0.0384	**0.7279±0.0788**
	Dataset 4	0.7415±0.0543	0.7526±0.0770	0.7501±0.0500	0.7259±0.0613	0.0478±0.0187	0.6351±0.0546	**0.8871±0.0385**
	Dataset 5	0.7600±0.1657	0.7927±0.0561	0.8535±0.0470	0.8026±0.0394	0.1224±0.0356	0.8926±0.0292	**0.8992±0.0218**
	Ave.	0.6977	0.7755	0.6972	0.6643	0.0483	0.4075	**0.8264**
