# Supplementary material for: LPI-HyADBS: a hybrid framework for lncRNA-protein interaction prediction integrating feature selection and classification
Source: BMC Bioinformatics. 2021 Nov 26;22:568. doi: 10.1186/s12859-021-04485-x (PMC8620196; doi:10.1186/s12859-021-04485-x)
Supplement: Supplementary file 3 — Additional file 3: Table SIII. The performance of seven LPI prediction methods on CVlp, the precision, recall, accuracy, F1-score, AUC and AUPR values obtained from LPI-SKF, LPI-NRLMF, Capsule-LPI, LPI-CNNCP, LPLNP, LPBNI, and LPI-HyADBS on five datasets under CVlp. [file 12859_2021_4485_MOESM3_ESM.pdf]

Table III The performance of seven LPI prediction methods on CV_{lp}

Metric	Dataset	LPI-SKF	LPI-NRLMF	Capsule-LPI	LPI-CNNCP	LPLNP	LPBNI	LPI-HyADBS
Precision	Dataset 1	0.7979±0.0337	0.8252±0.0107	0.8473±0.0112	0.7636±0.1614	0.4448±0.0738	0.5448±0.0542	**0.8654±0.0123**
	Dataset 2	0.7902±0.0059	0.8420±0.0081	0.8495±0.0094	0.7647±0.2231	0.4689±0.0944	0.5537±0.0883	**0.8762±0.0130**
	Dataset 3	**0.7631±0.0095**	0.7333±0.0077	0.7183±0.0152	0.6415±0.2759	0.5211±0.0571	0.7500±0.0354	0.7592±0.0125
	Dataset 4	0.7948±0.0070	0.7591±0.0144	0.7586±0.0241	0.5710±0.2977	0.6469±0.0259	0.7575±0.0662	**0.8413±0.0269**
	Dataset 5	0.8248±0.0011	0.8133±0.0043	0.8685±0.0076	0.7458±0.0986	0.7666±0.0025	0.8661±0.0468	**0.8903±0.0055**
	Ave.	0.7942	0.7945	0.8084	0.6973	0.5697	0.6944	**0.8465**
Recall	Dataset 1	0.9379±0.0283	0.7623±0.0162	**0.9532±0.0113**	0.7905±0.2850	0.4590±0.0934	0.2439±0.1292	0.9406±0.0101
	Dataset 2	0.6910±0.0092	0.7893±0.0174	0.9525±0.0118	0.7048±0.3640	0.4508±0.0516	0.1645±0.0412	**0.9565±0.0101**
	Dataset 3	0.6745±0.0065	0.6810±0.0132	**0.8316±0.0258**	0.5001±0.4367	0.4069±0.0291	0.0525±0.0391	0.8192±0.0194
	Dataset 4	0.7007±0.0052	0.7041±0.0307	0.8520±0.0319	0.5437±0.4280	0.5949±0.0391	0.4143±0.0354	**0.8561±0.0291**
	Dataset 5	0.7304±0.0006	0.8134±0.0050	0.8890±0.0104	0.8049±0.2272	0.7171±0.0055	0.6716±0.0215	**0.9219±0.0058**
	Ave.	0.7469	0.7500	0.8956	0.6688	0.5257	0.3094	**0.8989**
Accuracy	Dataset 1	0.8488±0.0136	0.7726±0.0115	0.8906±0.0076	0.7161±0.1420	**0.9845±0.0030**	0.9389±0.0251	0.8970±0.0075
	Dataset 2	0.6965±0.0057	0.7948±0.0096	0.8918±0.0077	0.7056±0.1415	**0.9902±0.0013**	0.9592±0.0006	0.9106±0.0094
	Dataset 3	0.6745±0.0065	0.6812±0.0078	0.7524±0.0117	0.5589±0.0725	**0.9652±0.0033**	0.8453±0.0018	0.7795±0.0101
	Dataset 4	0.7007±0.0052	0.7087±0.0160	0.7897±0.0176	0.5617±0.0800	**0.9546±0.0021**	0.8296±0.0038	0.8467±0.0185
	Dataset 5	0.7304±0.0006	0.7850±0.0044	0.8771±0.0031	0.7386±0.0826	**0.9588±0.0001**	0.8690±0.0089	0.9041±0.0029
	Ave.	0.7302	0.7484	0.8403	0.6561	**0.9707**	0.8884	0.8676
F1-score	Dataset 1	0.8614±0.0077	0.7924±0.0105	0.8971±0.0068	0.7098±0.2050	0.4397±0.0105	0.3370±0.0512	**0.9013±0.0068**
	Dataset 2	0.6565±0.0071	0.8147±0.0097	0.8980±0.0073	0.6450±0.2713	0.4515±0.0152	0.2536±0.2411	**0.9145±0.0087**
	Dataset 3	0.6359±0.0072	0.7061±0.0096	0.7705±0.0111	0.4126±0.2938	0.4540±0.0104	0.0981±0.2588	**0.7879±0.0103**
	Dataset 4	0.6636±0.0057	0.7303±0.0198	0.8020±0.0160	0.4394±0.3053	0.6187±0.0185	0.5357±0.0984	**0.8481±0.0180**
	Dataset 5	0.6923±0.0007	0.8133±0.0035	0.8785±0.0033	0.7365±0.1441	0.7409±0.0019	0.7566±0.0469	**0.9058±0.0027**
	Ave.	0.7019	0.7713	0.8492	0.5886	0.5410	0.3962	**0.8715**
AUC	Dataset 1	0.9293±0.0120	0.9222±0.0071	0.9380±0.0065	0.9197±0.0287	**0.9622±0.0019**	0.8459±0.0851	0.9488±0.0057
	Dataset 2	0.8893±0.0136	0.9401±0.0058	0.9404±0.0065	0.9210±0.0792	**0.9734±0.0013**	0.8306±0.0045	0.9583±0.0063
	Dataset 3	0.8493±0.0130	0.8138±0.0105	0.8343±0.0099	0.7500±0.1003	**0.9104±0.0057**	0.7518±0.0542	0.8593±0.0095
	Dataset 4	0.9024±0.0105	0.8501±0.0166	0.8518±0.0155	0.7677±0.0485	**0.9370±0.0064**	0.8228±0.0085	0.9162±0.0155
	Dataset 5	0.9609±0.0013	0.9247±0.0031	0.9474±0.0020	0.8597±0.0359	**0.9735±0.0030**	0.9318±0.0063	0.9672±0.0014
	Ave.	0.9062	0.8901	0.9023	0.8436	**0.9513**	0.8366	0.9300
AUPR	Dataset 1	0.9290±0.0155	0.9115±0.0103	0.9121±0.0107	0.9014±0.0255	0.4459±0.0092	0.3997±0.0454	**0.9300±0.0090**
	Dataset 2	0.8956±0.0128	0.9358±0.0087	0.9139±0.0118	0.9122±0.0556	0.4610±0.0176	0.3184±0.0566	**0.9423±0.0103**
	Dataset 3	**0.8560±0.0162**	0.8035±0.0138	0.8084±0.0111	0.7268±0.1177	0.4592±0.0100	0.4317±0.0841	0.8354±0.0128
	Dataset 4	0.6683±0.0061	0.8467±0.0266	0.8181±0.0246	0.7571±0.0492	0.6349±0.0309	0.6698±0.0152	**0.9098±0.0188**
	Dataset 5	0.9596±0.0021	0.9294±0.0034	0.9409±0.0030	0.8382±0.0377	0.8207±0.0027	0.8694±0.0784	**0.9653±0.0016**
	Ave.	0.8617	0.8853	0.8786	0.8271	0.5643	0.5378	**0.9166**
