# Supplementary material for: LPI-HyADBS: a hybrid framework for lncRNA-protein interaction prediction integrating feature selection and classification
Source: BMC Bioinformatics. 2021 Nov 26;22:568. doi: 10.1186/s12859-021-04485-x (PMC8620196; doi:10.1186/s12859-021-04485-x)
Supplement: Supplementary file 4 — Additional file 4: Table SIV. The performance of seven LPI prediction methods on CVind, the precision, recall, accuracy, F1-score, AUC and AUPR values obtained from LPI-SKF, LPI-NRLMF, Capsule-LPI, LPI-CNNCP, LPLNP, LPBNI, and LPI-HyADBS on five datasets under CVind. [file 12859_2021_4485_MOESM4_ESM.pdf]

**Table IV The performance of seven LPI prediction methods on $CV_{ind}$**

| Metric | Dataset | LPI-SKF | LPI-NRLMF | Capsule-LPI | LPI-CNNCP | LPLNP | LPBNI | LPI-HyADBS |
|---|---|---|---|---|---|---|---|---|
| Precision | Dataset 1 | 0.6534±0.1787 | 0.4990±0.0517 | 0.6016±0.1719 | 0.4154±0.3043 | 0.3219±0.2531 | 0.7348±0.0105 | **0.7753±0.1566** |
| | Dataset 2 | 0.6684±0.1303 | 0.4895±0.0445 | 0.6375±0.1462 | 0.3342±0.3431 | 0.3051±0.1757 | 0.3628±0.0654 | **0.7565±0.1928** |
| | Dataset 3 | **0.6400±0.1140** | 0.4976±0.0257 | 0.5475±0.0806 | 0.4206±0.2664 | 0.1909±0.1555 | 0.3823±0.0332 | 0.5764±0.1017 |
| | Dataset 4 | 0.4520±0.1099 | 0.4619±0.0724 | 0.5392±0.0745 | 0.3851±0.2177 | 0.1967±0.1301 | **0.8000±0.0138** | 0.5435±0.1405 |
| | Dataset 5 | 0.5625±0.1255 | 0.4984±0.0227 | 0.7568±0.0385 | 0.5969±0.2309 | 0.0937±0.0251 | **0.8598±0.0815** | 0.7797±0.0338 |
| | Ave. | 0.5953 | 0.4893 | 0.6165 | 0.4304 | 0.2217 | 0.6279 | **0.6863** |
| Recall | Dataset 1 | **0.5752±0.0986** | 0.4908±0.0365 | 0.5341±0.2858 | 0.4142±0.4270 | 0.4967±0.1780 | 0.3464±0.0541 | 0.4069±0.2227 |
| | Dataset 2 | **0.5709±0.0949** | 0.4991±0.0284 | 0.4713±0.2498 | 0.3450±0.4131 | 0.3889±0.3022 | 0.2603±0.0245 | 0.3701±0.2088 |
| | Dataset 3 | **0.5731±0.0896** | 0.4983±0.0195 | 0.5429±0.2039 | 0.4278±0.4253 | 0.4183±0.1530 | 0.4482±0.0213 | 0.3784±0.1623 |
| | Dataset 4 | 0.4689±0.0599 | 0.4961±0.0561 | 0.4894±0.1517 | **0.6442±0.4360** | 0.5988±0.1768 | 0.2181±0.0561 | 0.3706±0.1770 |
| | Dataset 5 | 0.4850±0.1105 | 0.4995±0.0149 | 0.6364±0.1012 | 0.6885±0.3048 | **0.8026±0.0511** | 0.7732±0.0519 | 0.6937±0.0787 |
| | Ave. | 0.5346 | 0.4967 | 0.5348 | 0.5039 | **0.5411** | 0.4092 | 0.4439 |
| Accuracy | Dataset 1 | 0.5774±0.1001 | 0.4998±0.0303 | 0.6234±0.1377 | 0.5440±0.0682 | **0.9902±0.0104** | 0.8940±0.0419 | 0.6539±0.1031 |
| | Dataset 2 | 0.5787±0.0990 | 0.5022±0.0250 | 0.6201±0.1133 | 0.5431±0.0718 | **0.9952±0.0061** | 0.8668±0.0068 | 0.6359±0.0906 |
| | Dataset 3 | 0.5723±0.0888 | 0.5016±0.0148 | 0.5508±0.0677 | 0.5249±0.0674 | **0.9695±0.0268** | 0.7757±0.0038 | 0.5564±0.0583 |
| | Dataset 4 | 0.4689±0.0599 | 0.4901±0.0428 | 0.5335±0.0589 | 0.5057±0.0160 | **0.9320±0.0686** | 0.6870±0.0067 | 0.5365±0.0738 |
| | Dataset 5 | 0.4850±0.1105 | 0.4993±0.0130 | 0.7134±0.0315 | 0.6654±0.0861 | **0.8698±0.0387** | 0.8621±0.0135 | 0.7479±0.0332 |
| | Ave. | 0.5365 | 0.4986 | 0.6082 | 0.5566 | **0.9513** | 0.8171 | 0.6261 |
| F1-score | Dataset 1 | 0.5417±0.1220 | 0.4940±0.0406 | **0.5461±0.2404** | 0.3484±0.3001 | 0.3317±0.1826 | 0.4708±0.0355 | 0.5049±0.2231 |
| | Dataset 2 | **0.5447±0.1013** | 0.4936±0.0333 | 0.5199±0.2094 | 0.2925±0.3169 | 0.2894±0.2274 | 0.3031±0.1512 | 0.4702±0.2137 |
| | Dataset 3 | **0.5374±0.0908** | 0.4977±0.0191 | 0.5269±0.1380 | 0.3505±0.2972 | 0.2224±0.1139 | 0.4126±0.0823 | 0.4417±0.1403 |
| | Dataset 4 | 0.4182±0.0677 | 0.4773±0.0620 | **0.5009±0.1006** | 0.4607±0.2823 | 0.2672±0.1121 | 0.3428±0.0359 | 0.4228±0.1588 |
| | Dataset 5 | 0.4540±0.1102 | 0.4989±0.0181 | 0.6855±0.0545 | 0.6224±0.2424 | 0.1672±0.0416 | **0.8142±0.0454** | 0.7313±0.0474 |
| | Ave. | 0.4992 | 0.4923 | **0.5558** | 0.4149 | 0.2556 | 0.4687 | 0.5142 |
| AUC | Dataset 1 | 0.6377±0.2467 | 0.5001±0.0623 | 0.6633±0.1690 | 0.6576±0.1283 | **0.9457±0.0222** | 0.9022±0.0124 | 0.8097±0.0959 |
| | Dataset 2 | 0.6529±0.1995 | 0.5071±0.0566 | 0.6619±0.1421 | 0.7126±0.1223 | **0.9645±0.0120** | 0.8441±0.0183 | 0.7935±0.0896 |
| | Dataset 3 | 0.6458±0.1790 | 0.5044±0.0298 | 0.5703±0.0864 | 0.5255±0.1215 | **0.8738±0.0193** | 0.7266±0.0215 | 0.6049±0.0957 |
| | Dataset 4 | 0.4364±0.1221 | 0.4783±0.0953 | 0.5472±0.0715 | 0.5453±0.0771 | **0.9077±0.0665** | 0.8315±0.0091 | 0.5596±0.0872 |
| | Dataset 5 | 0.4701±0.2215 | 0.4986±0.0259 | 0.8025±0.0263 | 0.7713±0.0540 | 0.8880±0.0255 | **0.9362±0.0039** | 0.8538±0.0281 |
| | Ave. | 0.5686 | 0.4977 | 0.6490 | 0.6424 | **0.9159** | 0.8481 | 0.7243 |
| AUPR | Dataset 1 | 0.7315±0.1836 | 0.5176±0.0576 | 0.6535±0.1485 | 0.6408±0.1095 | 0.1411±0.0821 | 0.6198±0.0199 | **0.7695±0.1009** |
| | Dataset 2 | 0.7305±0.1552 | 0.5078±0.0456 | 0.6440±0.1199 | 0.6948±0.1153 | 0.1484±0.1181 | 0.3212±0.0421 | **0.7549±0.0954** |
| | Dataset 3 | **0.6903±0.1582** | 0.5066±0.0249 | 0.5607±0.0658 | 0.5248±0.0972 | 0.0994±0.0618 | 0.4195±0.0848 | 0.5833±0.0752 |
| | Dataset 4 | 0.4768±0.0897 | 0.4940±0.0867 | 0.5521±0.0637 | 0.5465±0.0677 | 0.1225±0.0572 | **0.7054±0.0991** | 0.5718±0.0872 |
| | Dataset 5 | 0.6098±0.1591 | 0.5004±0.0229 | 0.7735±0.0339 | 0.7406±0.0366 | 0.0649±0.0181 | **0.9095±0.0026** | 0.8276±0.0365 |
| | Ave. | 0.6478 | 0.5052 | 0.6367 | 0.6295 | 0.1153 | 0.5951 | **0.7014** |
